# Rescue of alveolar wall liquid secretion blocks fatal lung injury due to influenza-staphylococcal coinfection

**DOI:** 10.1172/JCI163402

**Published:** 2023-10-02

**Authors:** Stephanie Tang, Ana Cassandra De Jesus, Deebly Chavez, Sayahi Suthakaran, Sarah K.L. Moore, Keshon Suthakaran, Sonya Homami, Raveen Rathnasinghe, Alison J. May, Michael Schotsaert, Clemente J. Britto, Jahar Bhattacharya, Jaime L. Hook

**Affiliations:** 1 Lung Imaging Laboratory, Division of Pulmonary, Critical Care, and Sleep Medicine, Department of Medicine,; 2 Graduate School of Biomedical Sciences,; 3 Global Health and Emerging Pathogens Institute, Department of Microbiology,; 4 Department of Cell, Developmental and Regenerative Biology,; 5 Department of Otolaryngology, and; 6 Institute of Regenerative Medicine, Icahn School of Medicine at Mount Sinai, New York, New York, USA.; 7 Division of Pulmonary, Critical Care, and Sleep Medicine, Department of Medicine, Yale University School of Medicine, New Haven, Connecticut, USA.; 8 Departments of Medicine and Physiology and Cellular Biophysics, College of Physicians and Surgeons, Columbia University Medical Center, New York, New York, USA.

**Keywords:** Pulmonology, Epithelial transport of ions and water, Influenza, Innate immunity

## Abstract

Secondary lung infection by inhaled *Staphylococcus aureus* (SA) is a common and lethal event for individuals infected with influenza A virus (IAV). How IAV disrupts host defense to promote SA infection in lung alveoli, where fatal lung injury occurs, is not known. We addressed this issue using real-time determinations of alveolar responses to IAV in live, intact, perfused lungs. Our findings show that IAV infection blocked defensive alveolar wall liquid (AWL) secretion and induced airspace liquid absorption, thereby reversing normal alveolar liquid dynamics and inhibiting alveolar clearance of inhaled SA. Loss of AWL secretion resulted from inhibition of the cystic fibrosis transmembrane conductance regulator (CFTR) ion channel in the alveolar epithelium, and airspace liquid absorption was caused by stimulation of the alveolar epithelial Na^+^ channel (ENaC). Loss of AWL secretion promoted alveolar stabilization of inhaled SA, but rescue of AWL secretion protected against alveolar SA stabilization and fatal SA-induced lung injury in IAV-infected mice. These findings reveal a central role for AWL secretion in alveolar defense against inhaled SA and identify AWL inhibition as a critical mechanism of IAV lung pathogenesis. AWL rescue may represent a new therapeutic approach for IAV-SA coinfection.

## Graphical abstract

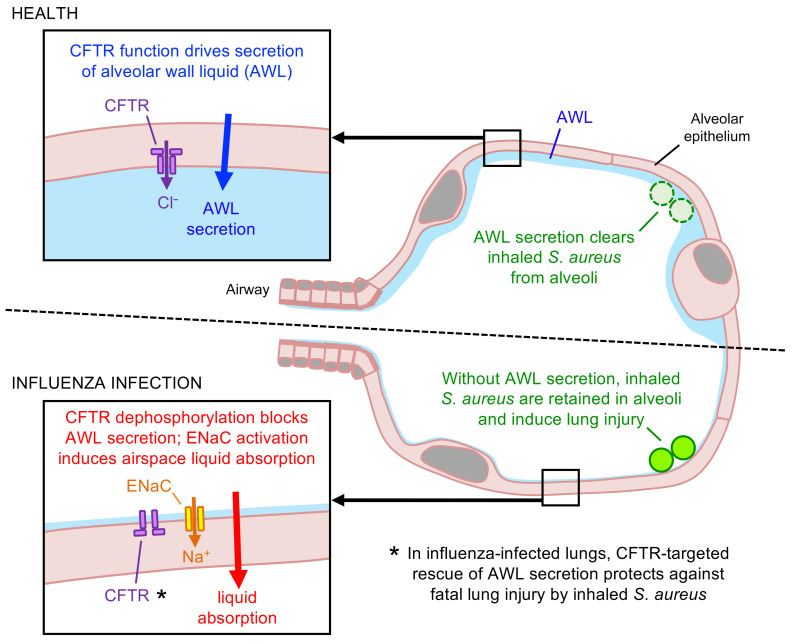

## Introduction

Lung infection is the fourth leading cause of global mortality (1), and the majority of severe lung infections are caused by respiratory viruses (2, 3). Among virus-infected individuals, the highest rates of death occur in those who acquire secondary lung infection by inhaled bacteria (4–9). Factors that account for the virulence of viral-bacterial coinfection remain unclear. Since coinfection pathogenesis centers on acute lung injury (10, 11), a disease of lung alveoli (12, 13), coinfection virulence might result from virus-induced alveolar responses that render alveoli susceptible to bacterial colonization and toxicity. However, there is little understanding as to how alveoli respond to respiratory viruses, including those responses that promote secondary bacterial infection. Here, we consider these issues in the context of lung coinfection by influenza A virus (IAV) and *Staphylococcus aureus* (SA), a common and lethal (4–7) pathogen combination for which current therapy is insufficiently effective (14) and increasingly hindered by pathogen drug resistance (15, 16).

Known mechanisms by which IAV promotes secondary SA infection focus on non-alveolar aspects of the respiratory system: airways, microvessels, and immune cells. Thus, SA inhaled into IAV-infected lungs encounter IAV-induced airway epithelial changes that promote bacterial adherence, including cell loss that exposes bacterial attachment sites (17) and impaired mucociliary velocity that hampers bacterial clearance (18). Survival of inhaled SA is enhanced by IAV-induced immune cell dysfunction that impairs bacterial uptake and killing (19, 20). Subsequently, SA cause acute lung injury by inducing exuberant inflammatory cell recruitment (21, 22), innate immune cell necrosis (23), and microvascular endothelial barrier dysfunction (24). Although these mechanisms address major aspects of coinfection pathogenesis, they do not address how IAV promotes SA infection in alveoli. This knowledge gap is important because alveoli comprise more than 95% of the lung surface area (25, 26) and are the site of the epithelial barrier dysfunction that drives fatal SA-induced lung injury (27).

IAV might promote SA infection in alveoli by disrupting alveolar defense. Alveolar defense mechanisms include bacterial killing by surfactants and alveolar macrophages (28–30) and particle removal by alveolar wall liquid (AWL) flow (31). AWL flow is generated on the alveolar surface by epithelial AWL secretion and convectively transports particles (31) — and perhaps SA (27) — out of alveoli. Since AWL secretion depends on function of the alveolar epithelial cystic fibrosis transmembrane conductance regulator (CFTR) protein (31), an ion channel inhibited by IAV in vitro (32–34), we considered that IAV might promote alveolar SA infection by blocking defensive, CFTR-dependent AWL secretion.

We tested this hypothesis by carrying out what we believe to be the first determinations of alveolar responses to IAV lung infection in live, intact, perfused lungs. Our findings show that IAV did indeed block AWL secretion by CFTR inhibition. However, IAV also caused airspace liquid absorption through activation of the alveolar epithelial Na^+^ channel (ENaC). The outcome was a remarkable reversal of normal alveolar liquid dynamics that abrogated AWL secretion, thereby promoting the alveolar stabilization of inhaled SA. Rescue of AWL secretion in IAV-infected mice restored alveolar SA clearance and protected against SA-induced lung injury. These findings show, for the first time to our knowledge, that AWL secretion contributes critically to lung defense against SA. Its restoration in IAV-infected lungs may represent a new therapeutic approach for the prevention of fatal SA coinfection.

## Results

### IAV lung infection blocks AWL secretion and induces alveolar liquid absorption.

**Figure 1:**
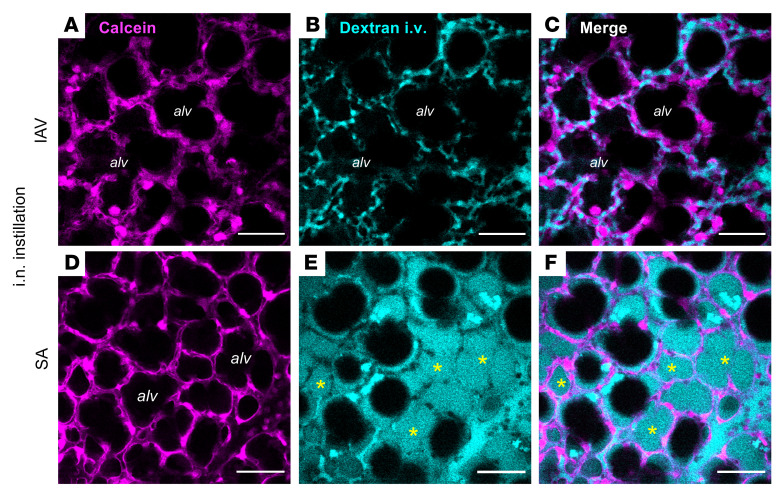
Alveolar epithelial viability and barrier function in live, intact, perfused lungs. (**A**–**F**) Confocal images show epithelial fluorescence of calcein (magenta) and intravascular (i.v.) fluorescence of tetramethylrhodamine-labeled dextran (20 kDa; 10 mg/mL; cyan) in live alveoli of intact, blood-perfused mouse lungs. Lungs were excised for imaging at 24 hours after intranasal (i.n.) IAV instillation (**A**–**C**) or 4 hours after intranasal SA instillation (**D**–**F**). Calcein-AM was microinstilled in alveoli by alveolar micropuncture, and dextran was added to the lung perfusate solution. Example dextran-filled airspaces are indicated by asterisks (**E** and **F**). Note that dextran fluorescence fills numerous alveolar airspaces (alv) in the SA-infected lung (**E** and **F**) but is absent from airspaces in the IAV-infected lung (**B** and **C**). Bacteria are not shown. Scale bars: 50 μm. Each set of images was replicated in lungs of 3 mice.

We used our established methods (27) to view live alveoli of intact, perfused mouse lungs by real-time confocal microscopy. Mice were untreated or intranasally instilled with IAV or SA at 24 hours and 4 hours, respectively, prior to lung excision for imaging. We determined epithelial viability by microinstillation of calcein dye into alveolar airspaces, then defined barrier function by addition of fluorophore-labeled dextran (20 kDa) to the lung perfusate solution. In IAV-infected lungs, cytosolic calcein fluorescence (Figure 1A) indicates that the alveolar epithelium was viable, and confinement of dextran fluorescence to microvessels of calcein-loaded alveoli (Figure 1, B and C) signals that barrier function was intact. Although the epithelium was also viable in SA-infected lungs (Figure 1D), dextran leak into airspaces (Figure 1, E and F) indicates that barrier dysfunction caused edema formation, aligning with our published data (27). We conclude that the alveolar epithelium retained viability and barrier function at 24 hours after IAV infection.

**Figure 2:**
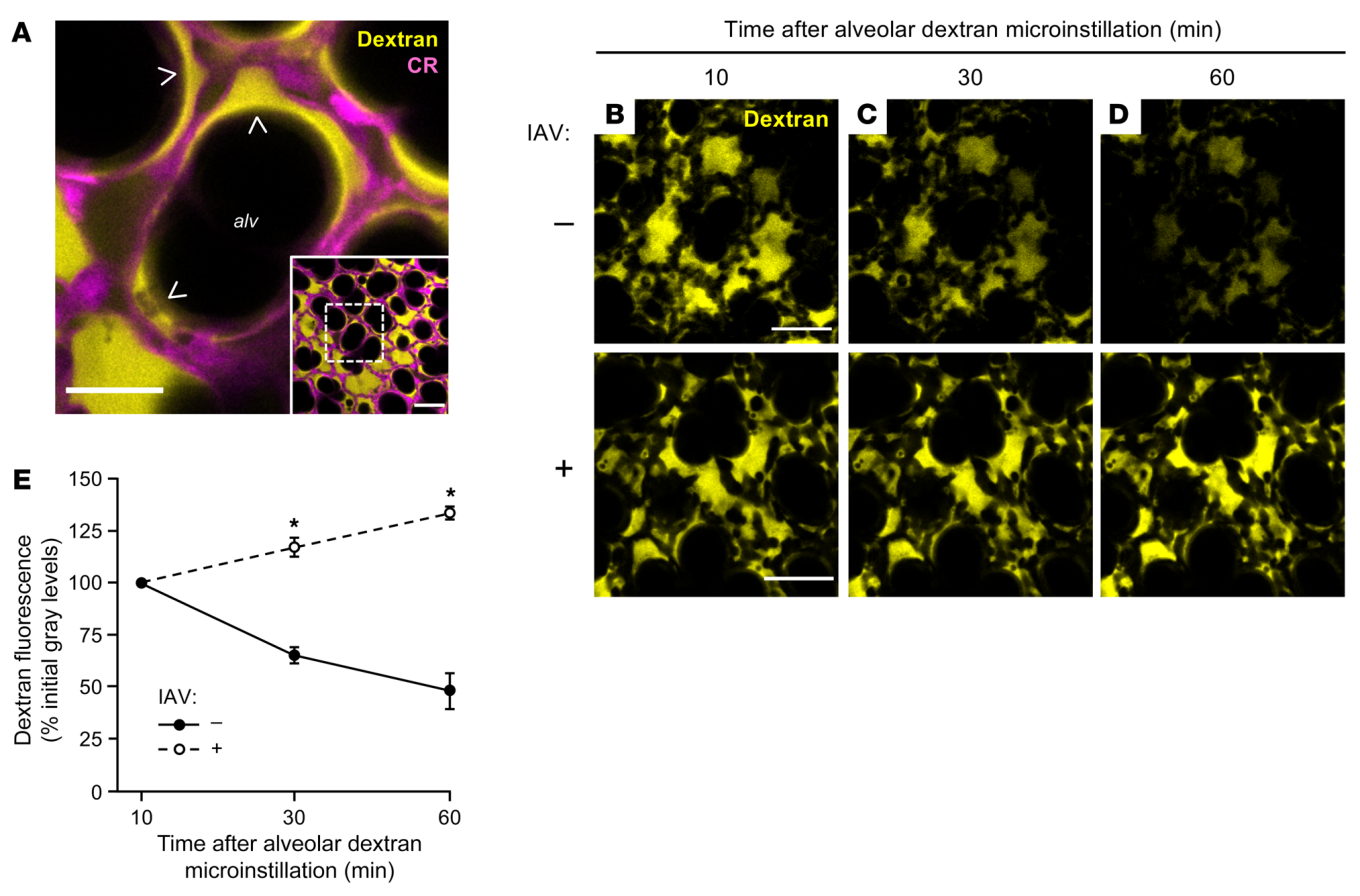
IAV lung infection disrupts AWL secretion in live alveoli. (**A**) Low-power (inset) and high-power confocal images show fluorescence of tetramethylrhodamine-conjugated (TRITC-conjugated) dextran (70 kDa; 10 mg/mL; yellow) in live alveoli (magenta) at 10 minutes after alveolar dextran microinstillation. Note that dextran formed a thin layer against alveolar walls and pooled in structural alveolar niches (arrowheads). CR, calcein red-orange; alv, alveolar airspace. Scale bars: 50 (inset) and 20 μm. Images replicated in 40 mice. (**B**–**E**) Confocal images (**B**–**D**) and group data (**E**) show time-dependent change of alveolar dextran fluorescence in airspaces of live alveoli in lungs excised from mice that were untreated (**B**–**D**), top row, and **E**, filled circles; *n* = 4 mice) or intranasally instilled with IAV at 24 hours before imaging (**B**–**D**, bottom row, and **E**, open circles; *n* = 4 mice). Fluorescence of alveolar walls is not shown. Group data (**E**) represent mean ± SEM. For each mouse, mean dextran fluorescence was quantified at each of the 3 indicated time points in an imaging field containing at least 30 lung alveoli. *P < 0.05 vs. closed circles by 2-tailed *t* test. Scale bars: 50 μm.

To visualize the AWL, we used an established approach (27, 31) in which we perfused the lungs with non-fluorescent blood-buffer solution, then micropunctured single alveoli under bright-field microscopy to instill alveolar airspaces with a 2-second microinfusion of fluorophore-labeled dextran (70 kDa) in aqueous solution. The microinfusion spread to airspaces of at least 20 neighboring alveoli, as evidenced by transient loss of optical discrimination between alveolar walls and airspaces. Return of optical discrimination occurred within seconds of each microinstillation, indicating that free fluid rapidly drained from alveoli and reestablished the air-filled alveolar lumens (35). In line with published findings from our group (31), confocal imaging revealed dextran fluorescence in airspaces as a juxtaepithelial layer that accumulated at alveolar niches (Figure 2A, arrowheads), curved regions of alveolar walls where septa converge (27). Airspace washout by alveolar microinfusion of non-fluorescent buffer abolished the dextran fluorescence (data not shown), indicating that dextran was restricted to airspaces and not taken up by the alveolar wall.

**Figure 3:**
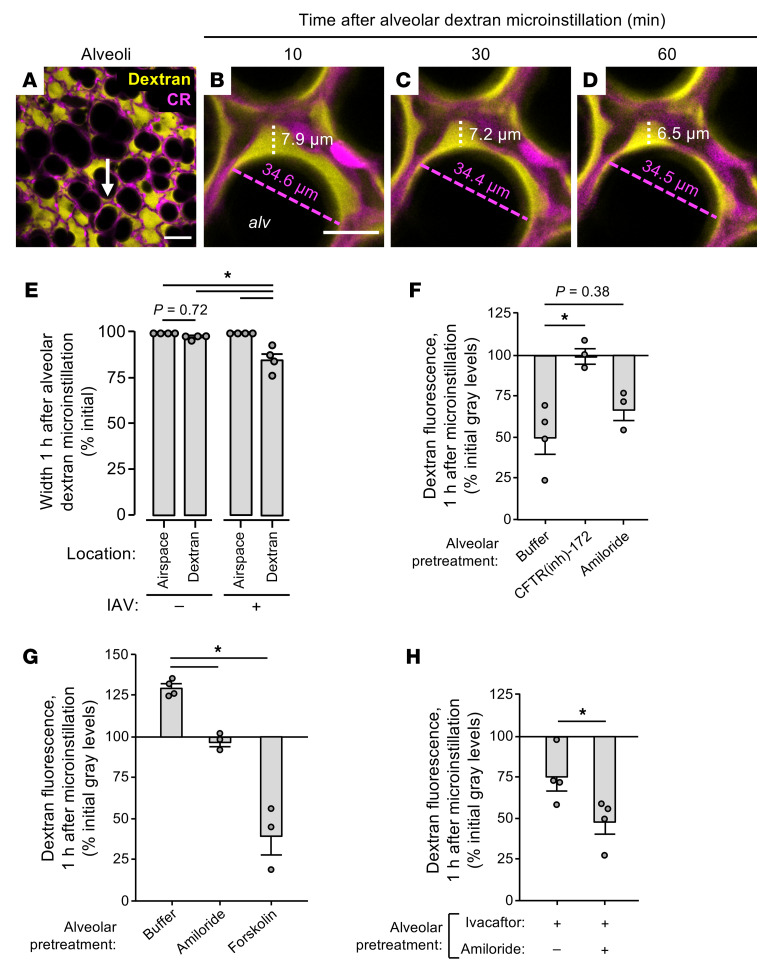
IAV lung infection induces airspace liquid absorption in live alveoli. (**A**–**E**) Confocal images (**A**–**D**) and group data (**E**) show time-dependent change of fluorescence of microinstilled TRITC-conjugated dextran (70 kDa; 10 mg/mL; yellow) in the live alveolus (magenta) shown in Figure 2A. Imaged lungs were excised from mice that were untreated (“–”; shown in **E** only) or intranasally instilled with IAV (“+”; **A**–**E**) at 24 hours before excision. High-power confocal views (**B**–**D**) of the structural alveolar niche (**A**, arrow) demonstrate, in IAV-infected lungs, time-dependent decrease of dextran pool width (white dashed lines and text) but not airspace width (magenta dashed lines and text). For group data (**E**), circles indicate *n* and each represent 1 mouse in which widths were quantified at 10 random locations in an imaging field containing at least 30 alveoli. Bars represent mean ± SEM; *P < 0.05 as indicated by ANOVA with post hoc Tukey testing. CR, calcein red-orange; alv, alveolar airspace. Scale bars: 50 (**A**) and 15 (**B**) μm. (**F**–**H**) Group data quantify change of TRITC-dextran fluorescence in alveolar airspaces of live, intact lungs. Mice were untreated (**F**) or intranasally instilled with IAV (**G** and **H**) at 24 hours before lung excision for imaging. The alveolar epithelium was pretreated as indicated with alveolar microinstillation of HEPES-buffered solution (Buffer) or the indicated reagents dissolved in HEPES-buffered solution; then alveolar airspaces were microinstilled with dextran. Circles indicate *n* and each represent 1 mouse in which mean dextran fluorescence change was quantified in imaging fields of at least 30 alveoli. Note that dextran fluorescence increased in buffer-treated alveoli of IAV-infected lungs (**G**, first bar), suggesting that the dextran concentration increased over time. Bars represent mean ± SEM; *P < 0.05 as indicated by ANOVA with post hoc Tukey testing (**F** and **G**) or 2-tailed *t* test (**H**).

Calibration experiments in glass micropipettes showed that dextran fluorescence varied with dextran concentration (Supplemental Figure 1, A and B; supplemental material available online with this article; https://doi.org/10.1172/JCI163402DS1) and was unchanged after repeated imaging (data not shown). However, dextran fluorescence decreased over time in alveolar airspaces of unchallenged lungs (Figure 2, B–D, top row, and Figure 2E, filled circles), confirming published findings (31) and indicating that the dextran was progressively diluted by addition of non-fluorescent liquid. To determine whether the dilution resulted from airspace accumulation of airway liquid, we inferred time-dependent change of airspace dextran volume from quantifications of dextran pool width at alveolar niches in high-power images at a specific distance below the pleura (Figure 3, A–E). Our findings confirm our group’s published data (31) and show that airspace and dextran pool widths were steady in alveoli of unchallenged lungs (Figure 3E, first and second bars, respectively), indicating that there was no inflow of liquid from the airways during the period of dextran fluorescence loss. Hence, we interpret that airspace dextran dilution resulted from alveolar liquid secretion, confirming reports (31) that the alveolar epithelium continuously secretes AWL into alveolar airspaces under baseline conditions.

By contrast, time-dependent gain of airspace dextran fluorescence in alveoli of IAV-infected lungs (Figure 2, B–D, bottom row, and Figure 2E, open circles) indicates that dextran concentration progressively increased. At the same time, dextran pool width progressively decreased whereas airspace width was steady (Figure 3, A–D, white and magenta dashed lines, and Figure 3E, third and fourth bars), indicating that airspace liquid volume decreased during the period of dextran fluorescence gain. We interpret that IAV induced airspace liquid absorption. Equalization of airspace and microvascular dextran concentrations failed to abrogate the fluorescence gain (Supplemental Figure 2A), ruling out the possibility that the liquid absorption resulted from an osmotic gradient generated by our preparation. Persistence of the dextran fluorescence gain in lungs infected with IAV for 3 days (Supplemental Figure 2B) indicates that airspace liquid absorption was a sustained feature of IAV lung infection. Together, these findings show that IAV induced airspace liquid absorption in alveoli and suggest that it inhibited AWL secretion.

### IAV disrupts alveolar liquid dynamics through effects on CFTR and ENaC function.

To determine mechanisms, we repeated the dextran microinstillation experiments after alveolar epithelial exposure to pharmacologic activators and inhibitors of CFTR and ENaC, ion channels that respectively drive AWL secretion (31, 36) and lung liquid uptake (37, 38). First, we confirmed that baseline AWL secretion depends on alveolar epithelial CFTR function (31) by blocking airspace dextran fluorescence loss in untreated lungs with the CFTR inhibitor CFTRinh-172 (Figure 3F, middle bar). The ENaC inhibitor amiloride had no effect on dextran fluorescence loss (Figure 3F, right bar), confirming reports that ENaC function does not contribute to alveolar liquid dynamics under baseline conditions (31). By contrast, in IAV-infected lungs, amiloride abolished dextran fluorescence gain (Figure 3G, middle bar), indicating that ENaC activity drove IAV-induced airspace liquid absorption in alveoli. Restoration of dextran fluorescence loss in alveoli treated with the CFTR activator forskolin (Figure 3G, right bar) signaled that IAV blocked CFTR function to inhibit AWL secretion. Taking these findings together, we conclude that IAV lung infection had a dual effect on the alveolar epithelium characterized by ENaC activation and CFTR inhibition. The result was stimulation of airspace liquid absorption and loss of AWL secretion, leading to net liquid absorption in alveoli of IAV-infected lungs. Importantly, drug-induced activation of CFTR in the alveolar epithelium overcame the IAV effect to restore, hence “rescue,” AWL secretion in IAV-infected lungs.

Steady airspace dextran fluorescence in CFTR-inhibited alveoli of uninfected lungs (Figure 3F, middle bar) indicates that CFTR inhibition did not reveal or induce airspace liquid absorption under baseline conditions. These findings suggest that CFTR does not regulate ENaC function in the alveolar epithelium, though it may have a regulatory role in other epithelia (39). To determine the relationship between CFTR and ENaC function in IAV-infected lungs, we treated the alveolar epithelium with ivacaftor, a potentiator of human and murine CFTR (40), or ivacaftor and amiloride together. As expected, ivacaftor restored dextran fluorescence loss in IAV-infected lungs (Figure 3H, left bar), indicating that CFTR potentiation in the alveolar epithelium rescued AWL secretion. The ivacaftor-induced fluorescence loss was augmented by amiloride (Figure 3H, right bar), indicating that ENaC inhibition and CFTR potentiation had additive effects on the restoration of AWL secretion in IAV-infected lungs. These findings suggest that CFTR inhibition and ENaC activation occurred by separate mechanisms in IAV-infected lungs and support the notion that CFTR and ENaC function independently in the alveolar epithelium.

### IAV-induced loss of AWL secretion results from alveolar CFTR dephosphorylation.

**Figure 4:**
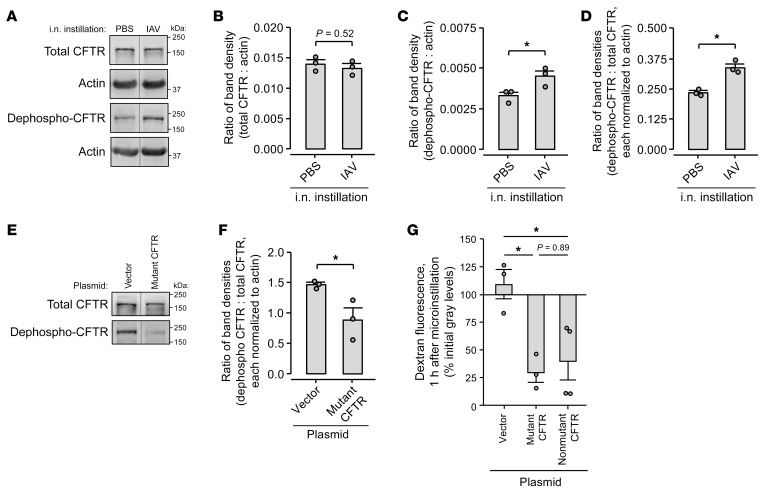
IAV lung infection causes CFTR dephosphorylation. (**A**–**D**) Lungs from mice intranasally instilled with IAV or PBS were excised at 24 hours after instillation and homogenized. Representative images (**A**) and group data of band densitometry (**B**–**D**) show immunoblot results using antibodies against total (clone A-3) and dephosphorylated (clone 570) CFTR protein as indicated. For group data (**B**–**D**), circles indicate *n* and each represent lungs of 1 mouse. Lanes were run on the same gel but were noncontiguous. *P < 0.05 by 2-tailed *t* test. (**E**–**G**) Mice were treated with (a) intranasal instillation of liposome-complexed plasmid DNA encoding the plasmid vector, A1440X mutant CFTR, or non-mutant CFTR; then (b) intranasal instillation of IAV at 24 hours. Lungs were excised at 48 hours after plasmid instillation for immunoblot (**E** and **F**) or imaging (**G**). In **E** and **F**, representative images (**E**) and group data of band densitometry (**F**) show immunoblot results using the indicated antibodies against total and dephosphorylated CFTR protein. Lanes were run on the same gel but were noncontiguous. Actin-probed membranes are not shown. In **F** and **G**, circles indicate *n* and each represent lungs of 1 mouse. In **G**, group data were derived by confocal imaging of live, intact, perfused mouse lungs and show change of TRITC-dextran fluorescence in alveolar airspaces after alveolar dextran microinstillation. Mean dextran fluorescence change was quantified in an imaging field of at least 30 alveoli. Bars represent mean ± SEM; *P < 0.05 by 1-tailed *t* test (**F**) or as indicated by ANOVA with post hoc Tukey testing (**G**).

Mechanisms of CFTR inhibition include protein degradation (32, 33) and dephosphorylation (41, 42). To define how IAV inhibited CFTR, we applied established methods of immunoblot quantification of total and dephosphorylated CFTR protein (41, 43–45) to whole-lung lysate. Immunoblots and band densitometry with and without actin normalization show that total lung CFTR protein content was equal in PBS- and IAV-instilled lungs at 24 hours after instillation (Figure 4, A and B, and Supplemental Figure 3A). However, density of the dephosphorylation-sensitive (41, 44) band was increased in lysate of IAV-instilled lungs, yielding an increased ratio of dephosphorylated CFTR to total CFTR band densities (Figure 4, A, C, and D, and Supplemental Figure 3, B and C). We interpret that lung content of dephosphorylated CFTR protein increased after IAV infection, whereas total CFTR protein content was unchanged. These findings indicate that IAV induced CFTR dephosphorylation in the lung within 24 hours of infection onset.

To evaluate whether CFTR dephosphorylation mediated IAV-induced AWL inhibition, we quantified AWL secretion in lungs pretreated with plasmid DNA encoding mutant, dephosphorylation-resistant CFTR protein (41, 46). The A1440X mutant CFTR contains a stop mutation at residue 1440, causing deletion of the 40 C-terminal amino acids, including major phosphatase binding sites (41, 46). Thus, although the mutant CFTR retains cell surface expression and Cl^–^ channel activity, it is dephosphorylated at a slow rate (41). After confirming the expected A1440X deletion by plasmid sequencing (data not shown), we transfected the alveolar epithelium with mutant CFTR or plasmid vector by intranasal instillation. We chose the intranasal route because our group has shown that intranasal plasmid instillation leads to plasmid expression in the alveolar epithelium (27, 47) and that the alveolar barrier blocks the trans-barrier spread of transfecting nucleotides (48, 49).

Our immunoblot findings show that alveolar epithelial transfection with mutant CFTR blocked CFTR dephosphorylation in IAV-infected lungs but had no effect on total CFTR protein in lungs that were either IAV-infected or uninfected (Figure 4, E and F, and Supplemental Figure 3, D–G). These findings indicate that mutant CFTR transfection functioned as expected to block IAV-induced CFTR dephosphorylation, but it did so without increasing total lung CFTR content, perhaps because of the known accelerated degradation of truncated CFTR mutants (50). Follow-up experiments using live lung imaging showed that mutant CFTR transfection induced airspace dextran fluorescence loss in alveoli of IAV-infected lungs (Figure 4G), indicating that alveolar epithelial expression of mutant CFTR protein disrupted the IAV effect to rescue AWL secretion. In control experiments, we transfected the alveolar epithelium with plasmid DNA encoding non-mutant CFTR, with the expectation that this transfection would fail to rescue AWL secretion. However, contrary to our expectation, non-mutant CFTR transfection also rescued AWL secretion in IAV-infected lungs (Figure 4G). Taking the imaging and immunoblot findings together, we interpret that IAV blocked AWL secretion by causing CFTR dephosphorylation in the alveolar epithelium. The IAV effect was disrupted by alveolar transfection with either mutant, dephosphorylation-resistant CFTR or non-mutant CFTR.

### IAV-induced loss of AWL secretion causes alveolar retention of inhaled SA.

**Figure 5:**
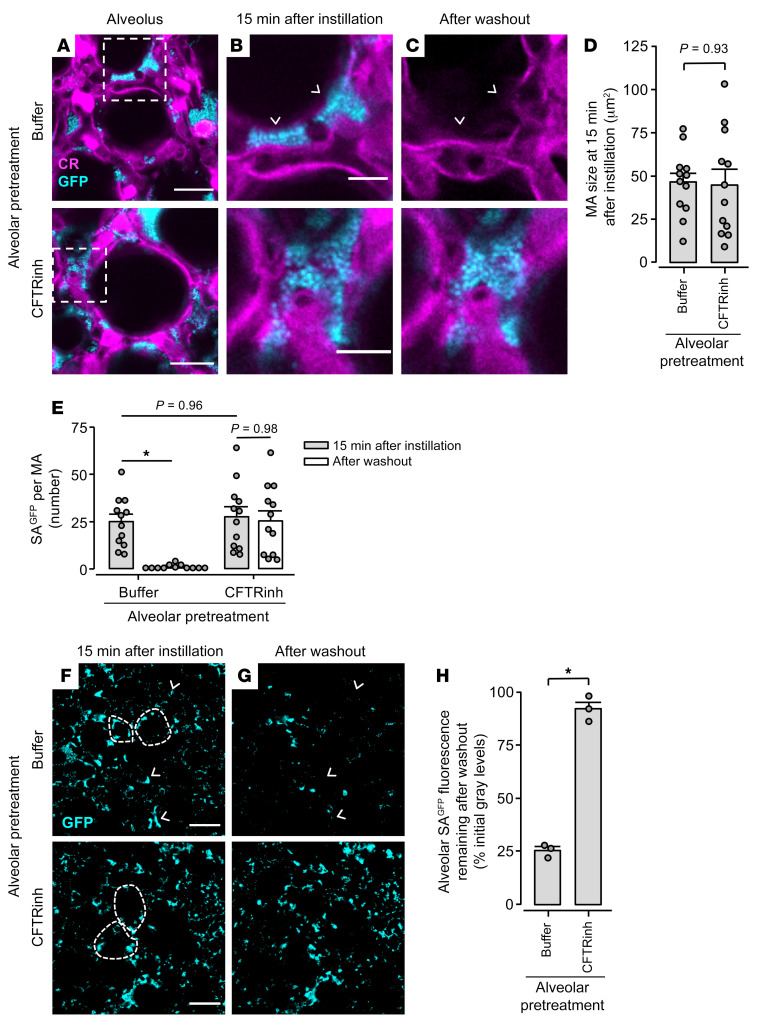
Alveolar epithelial CFTR function protects against alveolar stabilization of SA^GFP^. (**A**–**H**) High-power confocal images (**A**–**C**) and associated group data (**D** and **E**) and low-power images (**F** and **G**) and associated group data (**H**) show SA^GFP^ fluorescence in alveolar airspaces before and after alveolar washout. We pretreated alveoli with microinstillation of HEPES-buffered solution (Buffer) or CFTRinh-172 dissolved in HEPES-buffered solution, as indicated, then microinstilled alveolar airspaces with SA^GFP^. Alveoli were subjected to washout by vigorous alveolar microinstillation of buffer at 1 hour after SA^GFP^ microinstillation. Arrowheads (**B**, **C**, **F**, and **G**) point out example SA^GFP^ microaggregates (MA) that had complete loss of fluorescence in response to washout, hence were cleared from alveoli. In **A**, dashed squares indicate locations of images shown in **B** and **C**. In **F** and **G**, fluorescence of the alveolar epithelium is not shown, but dashed lines delineate example alveolar walls. Circles in **D**, **E**, and **H** indicate *n*. In **D** and **E**, circles each refer to one MA randomly selected before washout from 4 imaging fields of at least 30 alveoli. In **H**, circles were each generated by comparison of mean SA^GFP^ fluorescence before and after washout in 1 imaging field of at least 30 alveoli. Bars represent mean ± SEM; *P < 0.05 by 2-tailed *t* test (**D** and **H**) or as indicated by ANOVA with post hoc Tukey testing (**E**). Scale bars: 20 (**A**), 8 (**B**), and 50 (**F**) μm.

Our published data show that alveolar CFTR inhibition blocks spontaneous clearance of SA from alveoli (27), raising the possibility that loss of AWL secretion promotes alveolar SA stabilization. To test this possibility, we quantified the effect of AWL inhibition on alveolar stability of GFP-labeled SA in stationary growth phase (SA^GFP^). We selected the stationary growth phase because it may reflect the state of SA inhaled from the nasal cavity (51), since bacteria in stationary-like growth phases are prone to surface detachment (52). High-power images show that alveolar microinstillation of SA^GFP^ in alveoli pretreated with either buffer or CFTRinh-172 caused formation of SA^GFP^ microaggregates at alveolar niches (Figure 5, A–C). The microaggregates were of equal size (Figure 5D) and incorporated equal numbers of SA^GFP^ (Figure 5E, first and third bars), indicating that CFTR inhibition had no effect on microaggregate formation. Since microaggregate size (Figure 5D) exceeded the 6 μm tip opening diameter of the microinstillation pipettes, we rule out the possibility that the microaggregates formed prior to microinstillation. At 1 hour after microinstillation, we attempted to wash out the SA^GFP^ microaggregates by vigorous airspace buffer microinjection. Whereas washout caused loss of microaggregate fluorescence in buffer-pretreated alveoli (Figure 5, B and C, top images, and Figure 5E, second bar), it failed to clear microaggregates from CFTR-inhibited alveoli (Figure 5, B and C, bottom images, and Figure 5E, fourth bar). Low-power images affirmed the high-power findings (Figure 5, F–H). These data indicate that microaggregates in buffer-pretreated alveoli were susceptible to washout and, therefore, unstable against alveolar walls. However, microaggregates in CFTR-inhibited alveoli were highly stable and resisted dislodgement. We interpret that CFTR inhibition in the alveolar epithelium promoted stabilization of SA^GFP^ in alveoli. Thus, loss of alveolar epithelial CFTR function generated an alveolar microenvironment in which SA^GFP^ rapidly shifted phenotype from unstable to stabilized.

**Figure 6:**
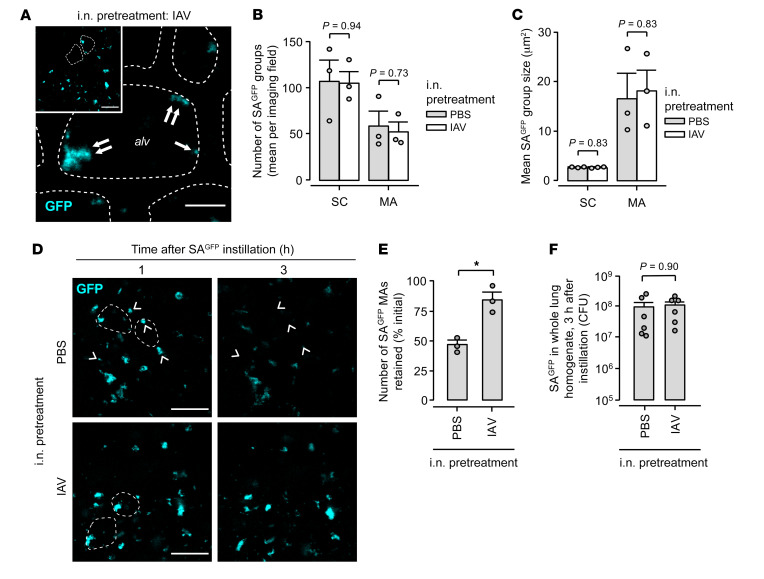
IAV lung infection causes alveolar retention of inhaled SA^GFP^. Mice were pretreated with intranasal instillation of IAV or PBS as indicated, then intranasally instilled with SA^GFP^ 24 hours later. For group data, circles indicate *n* and each represent 1 mouse. Bars represent mean ± SEM; *P < 0.05 as indicated by 2-tailed *t* test. (**A**–**C**) Low-power (inset) and high-power confocal images (**A**) show SA^GFP^ fluorescence in live alveoli of intact, blood-perfused, IAV-infected mouse lungs, 1 hour after intranasal SA^GFP^ instillation. Dashed lines delineate example alveolar walls (fluorescence not shown). Single and double arrows indicate SA^GFP^ grouped as small clusters (SC) and microaggregates (MA), respectively. Group data show number (**B**) and size (**C**) of SCs and MAs in alveoli of lungs pretreated with PBS or IAV instillation. For **B** and **C**, SA^GFP^ group number and size were quantified as means in at least 2 imaged fields of 30 alveoli each. Alv, example alveolar airspace. Scale bars: 50 (inset) and 10 μm. (**D** and **E**) Confocal images (**D**) show alveolar SA^GFP^ fluorescence at 1 hour (left) and, in the same alveoli, at 3 hours (right) after SA^GFP^ instillation. Arrowheads indicate example MAs that spontaneously lost all fluorescence, hence were cleared from alveoli. Group data (**E**) show the proportion of SA^GFP^ MAs that maintained alveolar fluorescence, hence were retained in alveoli. For **E**, MAs were quantified as the mean proportion retained in at least 2 imaged fields of 30 alveoli each. Scale bars: 50 μm. (**F**) Content of viable SA^GFP^ in lung homogenate at 3 hours after intranasal SA^GFP^ instillation.

Since IAV blocked alveolar epithelial CFTR function (Figure 3G), we considered that IAV might promote the stabilization of inhaled SA^GFP^ in alveoli. To test this hypothesis, we used live lung imaging to view alveoli after 2 intranasal instillations in mice: IAV or PBS, then, 24 hours later, SA^GFP^. Within 1 hour of instillation in IAV-infected lungs, SA^GFP^ formed microaggregates and small clusters in alveoli (Figure 6A). In line with our published findings (27), small clusters of non-microaggregated SA^GFP^ were present on flat alveolar surfaces (Figure 6A, single arrow), and microaggregates were located at alveolar niches (Figure 6A, inset and double arrows). Microaggregates and small clusters formed with equal frequency (Figure 6B) and size (Figure 6C) in alveoli of mice pretreated with IAV or PBS, indicating that the micromechanical features of alveoli that determine bacterial group formation were preserved in IAV-infected lungs. To determine the time course of spontaneous bacterial clearance, we imaged SA^GFP^-containing alveoli at 1 and 3 hours after SA^GFP^ instillation. Whereas most SA^GFP^ groups in PBS-pretreated lungs had complete loss of alveolar fluorescence (Figure 6D, top row, arrowheads, and Figure 6E, left bar), indicating that the bacteria were spontaneously removed from alveoli, the rate of alveolar SA^GFP^ fluorescence loss was markedly diminished in IAV-infected lungs (Figure 6D, bottom row, and Figure 6E, right bar), indicating that IAV caused failure of alveolar SA^GFP^ clearance. Together, these findings show that while IAV had no effect on niche-based microaggregate formation by inhaled SA^GFP^, it blocked alveolar SA^GFP^ clearance to promote SA^GFP^ retention in alveoli.

We considered that the retention might result from IAV-induced inhibition of SA killing (19, 20) or dissemination mechanisms. However, recovery of an equal number of viable SA^GFP^ from whole-lung homogenate of IAV- and PBS-pretreated mice (Figure 6F) indicates that SA killing was not impaired in IAV-infected lungs. Moreover, absence of CD11b^+^ cells in alveolar airspaces at 24 hours after IAV instillation and 3 hours after SA^GFP^ instillation (Supplemental Figure 4, A–E) rules out a role for neutrophils (53) — major effectors of SA killing — in the retention mechanism. To test whether the retention resulted from inhibition of SA dissemination, we quantified SA^GFP^ at extrapulmonary sites at 3 hours after SA^GFP^ instillation. Numbers of viable SA^GFP^ in blood, spleen, and liver were equal in mice pretreated with IAV and PBS (Supplemental Figure 5, A–C) and, in aggregate, represented less than 0.01% of the SA^GFP^ inoculum (Supplemental Figure 5D). These data indicate that inhibition of SA dissemination did not account for the retention. We interpret that failure of SA killing and dissemination were not mechanisms by which IAV caused alveolar SA^GFP^ retention.

**Figure 7:**
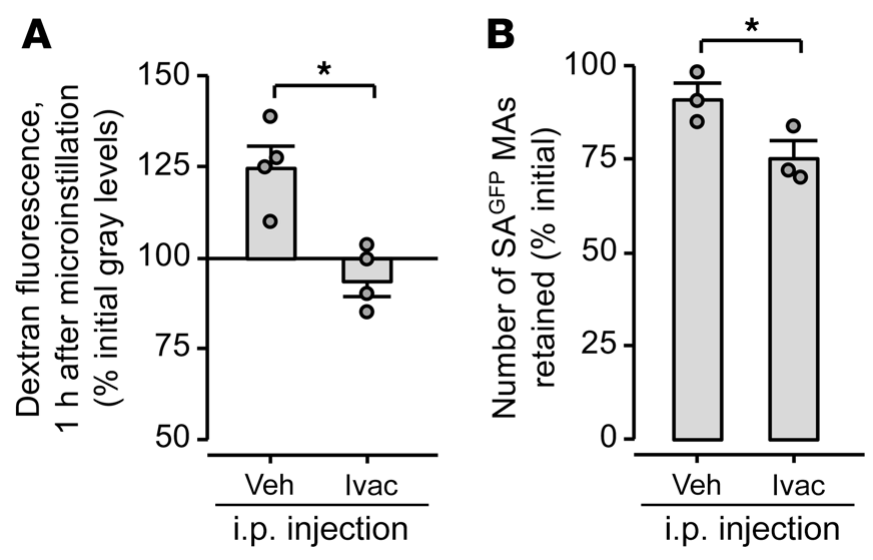
Systemic CFTR potentiation rescues AWL secretion and blocks alveolar SA^GFP^ stabilization in IAV-infected mice. Group data quantify confocal images of live, intact, perfused lungs. Mice were given intranasal instillation of IAV, then, at 6 hours, intraperitoneal injection of vehicle (Veh) or ivacaftor (Ivac) as indicated. (**A**) Lungs were excised for imaging at 24 hours after IAV instillation, and alveoli were microinstilled with TRITC-labeled dextran. Data show change of dextran fluorescence in alveolar airspaces. (**B**) At 24 hours after IAV instillation, mice were intranasally instilled with SA^GFP^, then the lungs were immediately excised for imaging. Data show spontaneous change of SA^GFP^ microaggregate (MA) fluorescence in alveolar airspaces from 1–3 hours after intranasal SA^GFP^ instillation. Circles indicate *n* and each represent 1 mouse in which change of dextran (**A**) or SA^GFP^ (**B**) fluorescence was quantified in imaging fields of at least 30 alveoli. Bars represent mean ± SEM; *P < 0.05 by 2-tailed *t* test.

Alternatively, the retention could have resulted from IAV-induced loss of CFTR-dependent AWL secretion, leading to loss of the alveolus-to-airway liquid flow that normally displaces particles from alveoli (31). To determine whether the retention resulted from CFTR inhibition, we evaluated alveolar SA^GFP^ retention in IAV-infected mice treated with the CFTR potentiator ivacaftor. We first affirmed that, like alveolar microinstillation of ivacaftor (Figure 3H), intraperitoneal injection of ivacaftor rescued AWL secretion in IAV-infected lungs (Figure 7A). These findings show that systemic ivacaftor administration restored CFTR function in alveoli. Next, we tested the effect of ivacaftor on alveolar retention of intranasally instilled SA^GFP^ in IAV-infected lungs. Ivacaftor blocked the retention (Figure 7B), indicating that CFTR inhibition was central to the retention mechanism.

Taking these findings together, we conclude that IAV-induced CFTR inhibition disrupted alveolar clearance of inhaled SA^GFP^, causing SA^GFP^ to assume a stabilized phenotype against alveolar walls. CFTR-targeted rescue of AWL secretion disrupted the stabilization to block alveolar SA^GFP^ retention and restore clearance of inhaled SA^GFP^ from alveoli.

### AWL rescue is protective in mouse models of IAV-SA coinfection.

**Figure 8:**
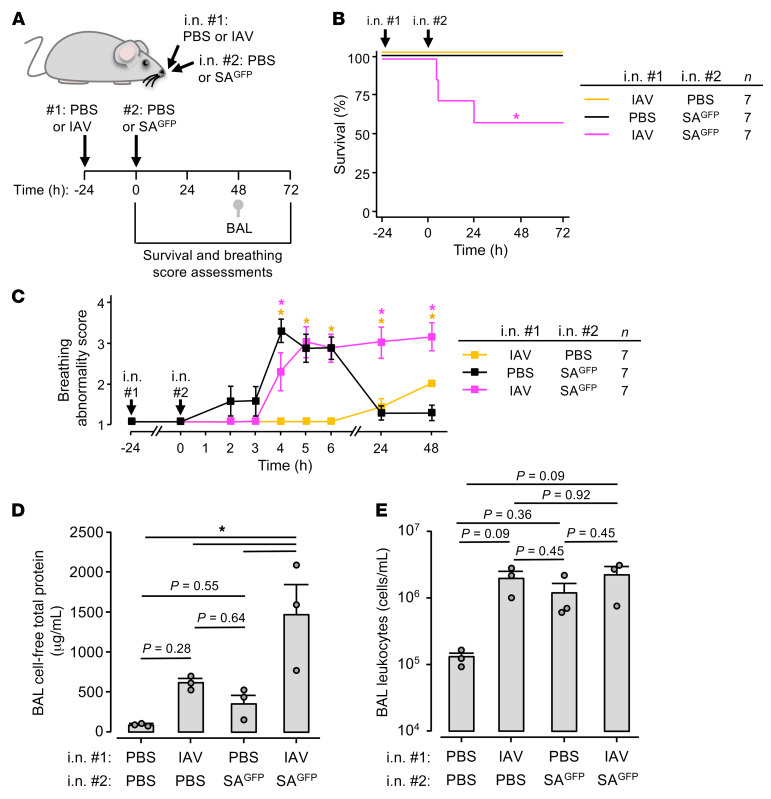
IAV augments the lung pathogenesis of SA^GFP^. Experimental design (**A**) for group data (**B**–**E**) indicates timing of intranasal instillations, survival (**B**) and breathing score (**C**) assessments, and BAL fluid collection for quantifications of total protein (**D**) and leukocytes (**E**). All mice were given a series of 2 instillations as indicated. Breathing scores (**C**) were imputed for non-surviving mice using their last observed value. Squares (**C**) and bars (**D** and **E**) indicate mean ± SEM; circles (**D** and **E**) indicate *n* and each represent data from 1 mouse; *P < 0.05 vs. black line by log rank (**B**) or 2-tailed *t* test (**C**) or as indicated by ANOVA with post hoc Tukey testing (**D** and **E**). BAL contents of protein (**D**) and leukocytes (**E**) were quantified using the same fluid specimens.

Our imaging findings show that IAV disrupted alveolar defense against inhaled SA^GFP^ within hours of IAV lung infection. To determine the extent to which the imaging data correlate with mouse models of lung infection, we compared mortality, lung injury, and lung inflammation in mice infected with IAV, SA^GFP^, and IAV and SA^GFP^ together. Mice were given 2 intranasal instillations, 24 hours apart (Figure 8A). Whereas mice instilled with IAV or SA^GFP^ alone each survived at least 3 days after the second instillation, mice coinfected with IAV and SA^GFP^ had nearly 50% mortality (Figure 8B), indicating that IAV augmented SA^GFP^ pathogenesis when SA^GFP^ was instilled 24 hours after IAV.

To determine mortality mechanisms, we assigned scores to the mice using an observational breathing score system (Supplemental Figure 6A) in which higher score correlated with higher bronchoalveolar lavage (BAL) fluid protein content (Supplemental Figure 6B), a marker of alveolar barrier dysfunction. Within hours of instillation, SA^GFP^ induced breathing abnormalities in mice pretreated with PBS or IAV (Figure 8C). Whereas the breathing abnormalities resolved within 24 hours in mice infected with SA^GFP^ alone (Figure 8C, black line), they persisted for days in coinfected mice (Figure 8C, magenta line). Mice infected with IAV alone had minimal breathing abnormalities during this period (Figure 8C, gold line). In line with the breathing scores, BAL protein content at 48 hours after SA^GFP^ instillation was increased only in coinfected mice (Figure 8D), indicating that IAV augmented SA^GFP^-induced alveolar barrier dysfunction. By contrast, BAL leukocyte content was equal across groups (Figure 8E). Taking these findings together, we interpret that IAV augmented SA^GFP^-induced lung injury and mortality in a mouse model in which IAV instillation preceded SA^GFP^ instillation by 24 hours.

**Figure 9:**
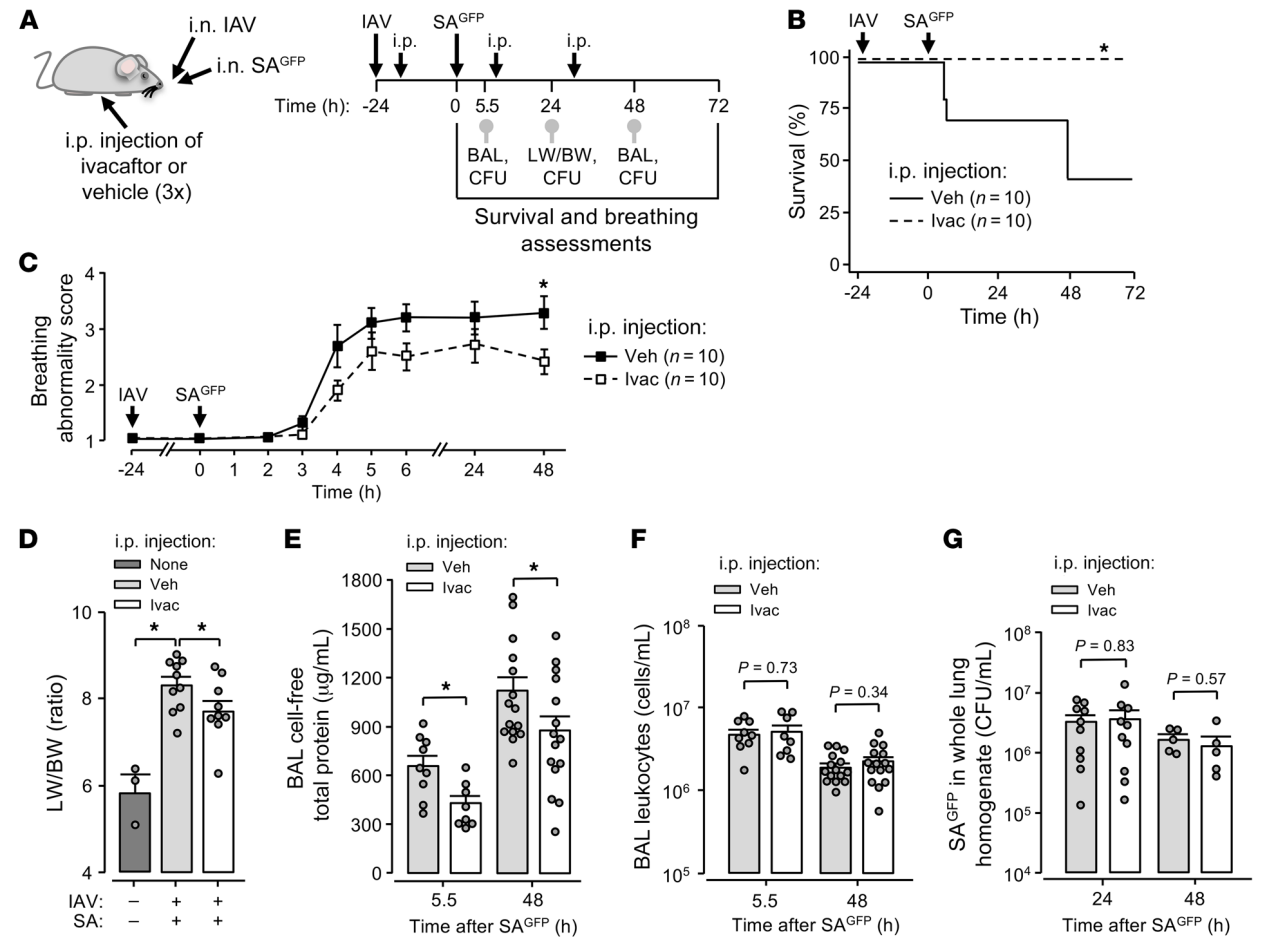
AWL rescue therapy protects against fatal IAV-SA^GFP^ coinfection. (**A**–**G**) Experimental design (**A**) for group data shown in **B**–**G** shows timing of intranasal instillations, intraperitoneal injections, and procedures including mouse survival (**B**) and breathing score (**C**) assessments, lung excision for quantification of lung wet weight to body weight (LW/BW) ratio (**D**), BAL fluid collection for quantification of total protein (**E**) and leukocyte (**F**) content, and lung excision for SA^GFP^ quantification (CFU; **G**). Note that 3 mice were untreated and are indicated in **D** (first bar). Breathing scores (**C**) were imputed for non-surviving mice using their last observed value. Squares (**C**) and bars (**D**–**G**) indicate mean ± SEM; circles (**D**–**G**) indicate *n* and each represent data from 1 mouse; *P < 0.05 vs. black line by log rank (**B**) or 2-tailed *t* test (**C**) or as indicated by 1- (**D**) or 2-tailed (**E**–**G**) *t* test. BAL contents of protein (**E**) and leukocytes (**F**) were quantified using the same fluid specimen.

We have shown previously that stabilization of inhaled SA in alveoli leads to SA-induced alveolar damage and fatal lung injury (27). We considered that, by restoring AWL secretion and blocking alveolar SA retention (Figure 7, A and B), AWL rescue therapy might protect against fatal SA-induced lung injury in IAV-infected mice. To test this possibility, we treated coinfected mice with systemic injection of vehicle or ivacaftor (Figure 9A). Whereas coinfected mice treated with vehicle had high mortality (Figure 9B, solid line), all coinfected mice treated with ivacaftor survived (Figure 9B, dashed line). These findings show that AWL rescue therapy protected against SA^GFP^-induced mortality in coinfected mice.

**Figure 10:**
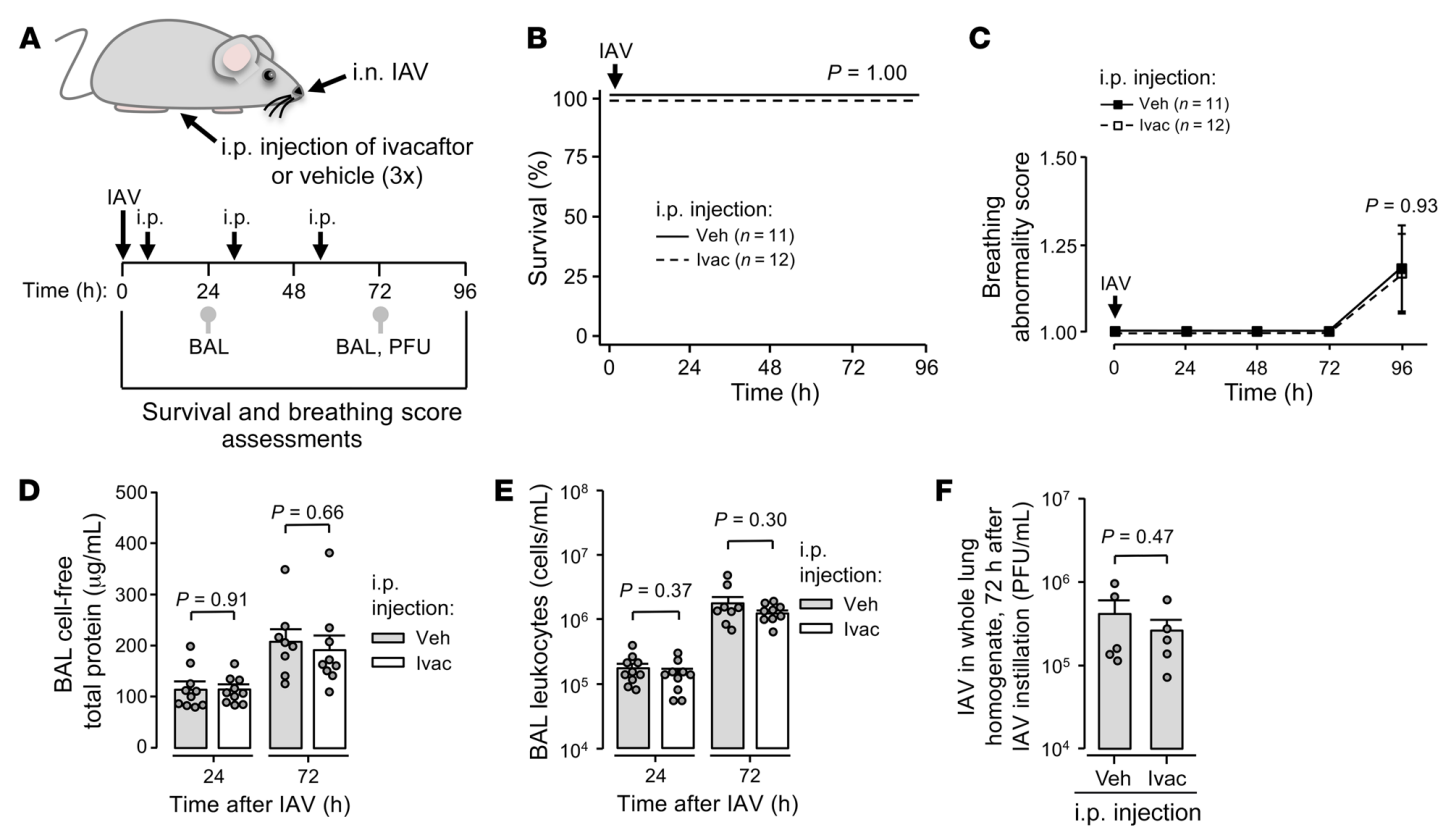
AWL rescue therapy does not affect early outcomes of IAV lung infection. (**A**–**F**) Experimental design (**A**) for group data shown in **B**–**F** shows timing of intranasal instillations, intraperitoneal injections, and procedures including mouse survival (**B**) and breathing score (**C**) assessments, BAL fluid collection for quantification of total protein (**D**) and leukocyte (**E**) content, and lung excision for IAV quantification (PFU; **F**). Breathing scores (**C**) were imputed for non-surviving mice using their last observed value. Squares (**C**) and bars (**D**–**F**) indicate mean ± SEM; circles (**D**–**F**) indicate *n* and each represent data from 1 mouse; P values were calculated vs. black line by log rank (**B**) or 2-tailed *t* test (**C**) or as indicated by 2-tailed *t* test (**D**–**F**). BAL contents of protein (**D**) and leukocytes (**E**) were quantified using the same BAL fluid specimen.

Next, we defined the extent to which ivacaftor’s survival benefit stemmed from protection against SA^GFP^-induced lung injury. Compared with coinfected mice treated with vehicle, mice treated with ivacaftor had lower breathing score, lung wet weight to body weight ratio, BAL protein content, and blood-free extravascular lung water content after SA^GFP^ instillation (Figure 9, C–E, and Supplemental Figure 7), indicating that AWL rescue therapy protected against SA^GFP^-induced lung injury in IAV-infected mice. To determine whether ivacaftor’s therapeutic benefit resulted from drug-induced leukocyte modulation (54) or SA killing (55), we quantified lung inflammation and SA burden (Figure 9A). Our findings show that ivacaftor had no effect on BAL leukocyte content or SA^GFP^ counts in whole-lung homogenate, BAL fluid, blood, spleen, or liver at multiple time points after SA^GFP^ instillation in IAV-infected mice (Figure 9, F and G, and Supplemental Figure 8, A–H), indicating that ivacaftor did not impact BAL-accessible lung inflammation, SA dissemination, or SA viability. In mice infected with IAV alone, equivalent survival, breathing score, BAL protein content, BAL leukocyte content, and lung IAV content between ivacaftor- and vehicle-treated groups (Figure 10, A–F) rule out the possibility that ivacaftor’s therapeutic effect in coinfected mice resulted from modulation of the primary IAV infection. Failure of ivacaftor to impact BAL protein or leukocyte content in mice infected with SA^GFP^ alone (Supplemental Figure 9, A–C) indicates that prior infection with IAV was required for ivacaftor to exert a therapeutic effect in the time frame of our experiments. Taking these findings together, we interpret that ivacaftor decreased SA^GFP^-induced mortality after IAV infection by protecting against lung responses to IAV that promoted SA^GFP^-induced lung injury. We conclude that systemic CFTR potentiation increased survival in coinfected mice by rescuing AWL secretion after IAV infection, in turn protecting against the alveolar stabilization of inhaled SA^GFP^ and mitigating SA^GFP^-induced lung injury.

**Figure 11:**
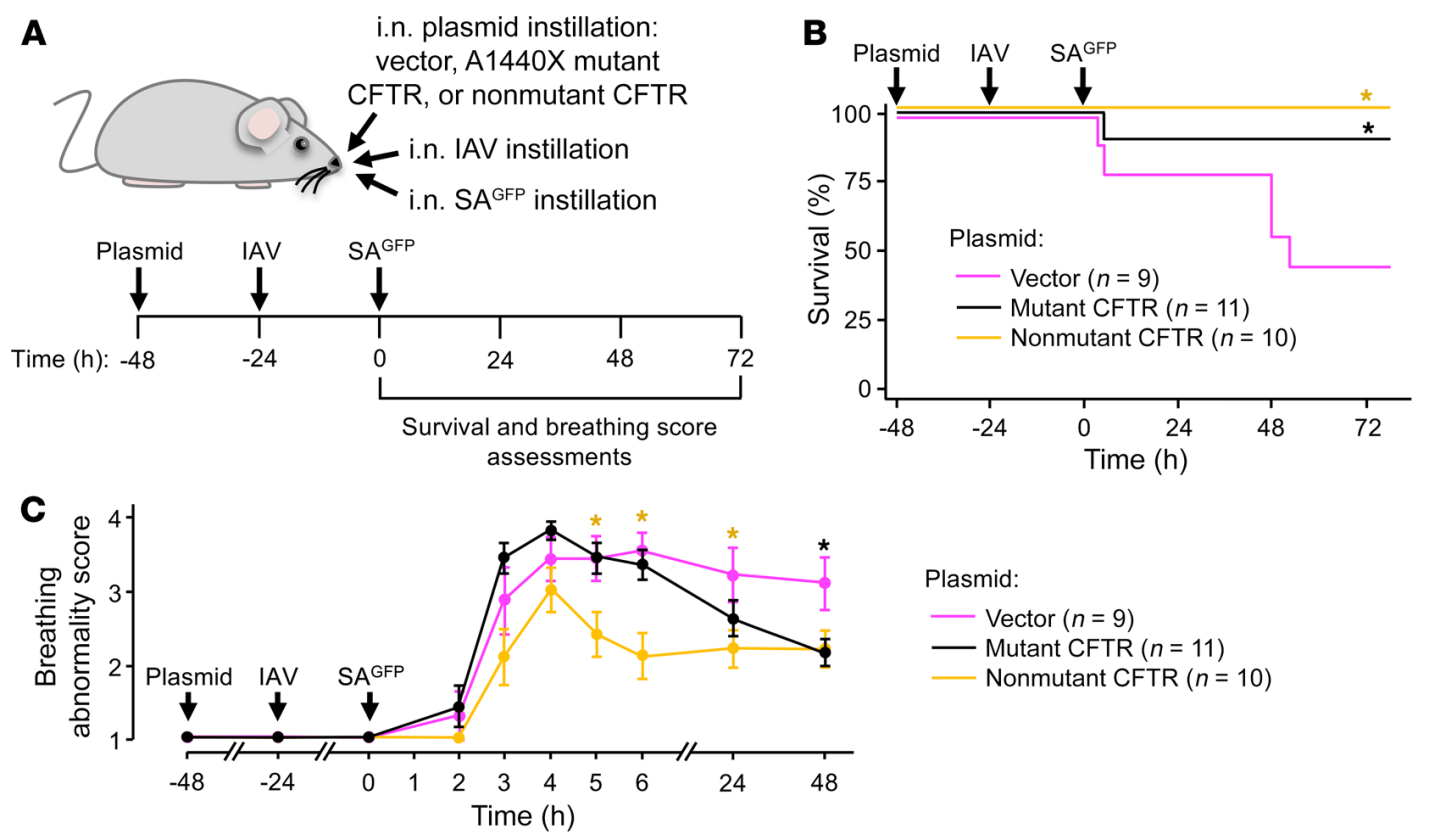
CFTR transfections protect against SA^GFP^-induced mortality in IAV-infected mice. Experimental design (**A**) for group data (**B** and **C**) indicates timing of intranasal instillations and survival (**B**) and breathing score (**C**) assessments. Breathing scores were imputed for non-surviving mice using their last observed value. Circles (**C**) indicate mean ± SEM; *n* as indicated; *P < 0.05 vs. magenta line by log rank testing (**B**) or 2-tailed *t* test (**C**).

It is not known whether CFTR dephosphorylation determines the pathogenicity of SA coinfection with IAV. To address this question, we pretreated mice with intranasal instillation of mutant, dephosphorylation-resistant CFTR plasmid, non-mutant CFTR plasmid, or plasmid vector. The pretreatment was followed by intranasal instillations of IAV, then SA^GFP^ (Figure 11A). Whereas vector-treated mice had high mortality (Figure 11B, magenta line), mice transfected with mutant CFTR had reduced mortality and breathing scores (Figure 11, B and C). Mice transfected with non-mutant CFTR also had reduced mortality and breathing scores (Figure 11, B and C). Together with the immunoblot and imaging data (Figure 4, E–G), these findings show that alveolar epithelial expression of mutant, dephosphorylation-resistant (41, 46) CFTR protein or non-mutant CFTR protein each rescued AWL secretion and protected against SA^GFP^-induced mortality in IAV-infected mice. We conclude that IAV-induced CFTR dephosphorylation in the alveolar epithelium was central to the lung pathogenesis of IAV-SA^GFP^ coinfection.

## Discussion

Our findings show, for the first time to our knowledge, that IAV lung infection caused reversal of alveolar liquid dynamics from epithelial secretion to absorption. The reversal occurred within hours of IAV instillation and resulted from 2 effects of IAV infection on the alveolar epithelium: CFTR inhibition, leading to loss of AWL secretion; and ENaC stimulation, causing alveolar liquid absorption. Alveolar epithelial treatment with CFTR activator or potentiator drugs rapidly restored AWL secretion in IAV-infected lungs, indicating that the electrochemical and osmotic gradients that normally drive AWL secretion were intact, and thus the AWL inhibition resulted directly from CFTR inhibition. The effect was the abrogation of a major mechanism of alveolar defense — namely, AWL secretion — to generate an alveolar microenvironment favorable to the stabilization of inhaled SA against alveolar walls. Thus, the new understanding gained from our findings is that AWL secretion is a major mechanism of innate alveolar defense against inhaled SA. Its inhibition by IAV lung infection promoted the initiation of SA infection in alveoli, leading to fatal SA-induced lung injury.

Since acute lung injury is traditionally defined by *gain* of airspace liquid, as pulmonary edema fluid (12, 13), our proposal that coinfection-induced lung injury resulted from *loss* of airspace liquid, as AWL, adds new understanding to lung injury mechanisms. Our findings show that IAV blocked AWL secretion, leading to retention of SA against alveolar walls for hours. The retention enabled SA, a pathogen known for its ability to rapidly adapt to its microenvironment (56), to transition from an unstable phenotype susceptible to alveolar clearance to a highly stable phenotype that resisted alveolar dislodgement. Thus, the alveolar microenvironment generated by IAV-induced loss of AWL secretion created an opportunity for SA to rapidly assume a stabilized phenotype that, we have shown previously, initiates alveolar infection and fatal alveolar damage (27). We interpret that AWL secretion contributes critically to alveolar defense against inhaled SA, and that AWL inhibition constitutes a major mechanism by which IAV promotes secondary SA infection.

How IAV-induced AWL inhibition caused alveolar SA retention remains unclear. Our findings show that CFTR inhibition in the alveolar epithelium did not affect formation of SA microaggregates but enabled their stabilization. Since our published data show that alveolar microaggregate stabilization results from SA interactions involving the SA surface protein PhnD (27), we propose that alveolar CFTR inhibition promoted the formation of stabilizing protein interactions among microaggregated SA. Such interactions might result from loss of CFTR-mediated HCO_3_^–^ secretion that could lower extracellular pH to a level favorable to SA self-adherence (57). At the same time, loss of CFTR-mediated, Cl^–^-driven AWL flow might block convective transport (31) of non-adherent SA out of alveoli. We rule out alternative mechanisms of alveolar SA retention, namely inhibition of SA killing or dissemination, by our findings that IAV had no effect on numbers of viable SA in the lungs or extrapulmonary organs. Although the mechanistic details remain uncertain, our finding that AWL rescue therapy restored alveolar SA clearance in IAV-infected lungs points to CFTR inhibition as a critical step in the retention mechanism.

Our data identify a central role for CFTR dephosphorylation in the alveolar pathogenesis of IAV lung infection. It is known that CFTR is expressed by alveolar epithelial type 1 (AT1) and 2 (AT2) cells (58–63) and drives alveolar epithelial secretion of Cl^–^ and liquid under baseline conditions (31, 36, 61, 64, 65). Here, we add that IAV blocked CFTR-mediated AWL secretion and induced CFTR dephosphorylation. Importantly, plasmid-mediated inhibition of CFTR dephosphorylation in the alveolar epithelium rescued AWL secretion in IAV-infected lungs, indicating that alveolar epithelial CFTR dephosphorylation was responsible for IAV-induced AWL inhibition. Thus, the efficacy of forskolin and ivacaftor for rescuing AWL secretion may have been related to their known capacity to potentiate CFTR function under non-phosphorylating conditions (66, 67). Together, these findings reveal a new mechanism of IAV-induced CFTR inhibition in the lung.

The capacity of non-mutant CFTR transfection to rescue AWL secretion and protect against SA-induced mortality in IAV-infected mice raises the possibility that dephosphorylation was not the sole mechanism by which IAV blocked CFTR function in the alveolar epithelium. For example, IAV might promote loss of CFTR protein in alveoli, in line with reports of IAV-induced CFTR ubiquitination and degradation in cultured airway epithelial cells (33, 34). Although we did not identify CFTR protein loss by immunoblot of whole-lung lysate after intranasal IAV instillation, the immunoblot may have failed to detect protein loss in alveoli if alveolar epithelial responses to IAV were masked by responses of non-alveolar cells. Alternatively, non-mutant CFTR transfection could have induced sufficient CFTR overexpression in the alveolar epithelium to overcome the inhibition caused by CFTR dephosphorylation, thereby rescuing AWL secretion in IAV-infected mice. These issues, as well as the upstream mechanisms responsible for IAV-induced CFTR dephosphorylation, warrant further study.

Our finding that IAV lung infection induced reversal of alveolar liquid dynamics — from basal AWL secretion to airspace liquid absorption — is, to our knowledge, novel, but it is well supported by existing literature. Our data demonstrating the presence of basal AWL secretion align with reports indicating that the intact alveolar epithelium secretes Na^+^ (36), Cl^–^ (31, 36, 64), and liquid (31, 36) under baseline conditions. Although it is known that AT1 and AT2 cells express ENaC (68–70) and exhibit rapid reversal from liquid secretion to absorption in response to local conditions (36, 61), our findings are the first, to our knowledge, to show that reversal from CFTR-mediated liquid secretion to ENaC-mediated liquid absorption occurred in the alveolar epithelium in response to IAV infection. The reversal of alveolar liquid dynamics had major bearing on the outcome of IAV-SA coinfection in that it promoted alveolar SA retention. While others have reported seemingly opposite findings, that IAV blocks lung absorption of airway-instilled liquid in rodents (71, 72), we point out that the contribution of the small airway epithelium to bulk transport of airway-instilled liquid is not understood. Importantly, our data provide direct evidence that IAV caused liquid absorption in airspaces of intact, perfused alveoli. Future research might build on our findings by defining whether AWL inhibition depends on direct IAV infection of alveoli and quantifying the extent to which AT1 cells, AT2 cells, and their subpopulations (73) determine liquid transport in perfused alveoli of IAV-infected and uninfected lungs. AT1 cells may drive alveolar liquid transport on account of their extensive surface area, which comprises more than 97% of the luminal alveolar surface (74).

An important aspect of our findings is the demonstration that IAV disrupted alveolar physiology in the first hours of lung infection. Although others have shown that IAV augments SA-induced lung pathogenesis when SA is instilled in mice at 3 days after IAV instillation (21, 22), there is limited understanding of lung responses to IAV and IAV-SA coinfection at earlier time points. Early alveolar responses to IAV that predispose to SA lung infection may have clinical relevance, since clinical data show that secondary bacterial infection may occur as early as 1–3 days after the onset of respiratory symptoms (6, 11, 75, 76) and cause symptoms indistinguishable from those of influenza alone (6, 77).

From a therapeutic perspective, our data suggest that AWL rescue represents a new therapeutic target for preventing fatal SA-induced lung injury after IAV infection. Although systemic ivacaftor injection induced only modest regain of AWL secretion in IAV-infected lungs, the gain was nevertheless sufficient to block alveolar stabilization of inhaled SA, perhaps because it was distributed across an extensive alveolar epithelial surface (25, 26). Subsequently, AWL rescue therapy decreased SA-induced lung injury and mortality in IAV-infected mice. Notably, ivacaftor decreased SA-induced alveolar barrier dysfunction without altering BAL-accessible lung leukocyte numbers or pathogen burden, placing alveolar responses to IAV and SA at the center of coinfection pathogenesis mechanisms and suggesting that protection against alveolar barrier dysfunction accounted for ivacaftor’s therapeutic effect. Although nonspecific mechanisms may have contributed, we propose that fatal SA-induced lung injury resulted from IAV-induced loss of AWL secretion, leading to alveolar retention of SA. Approaches that rescue AWL secretion in IAV-infected lungs may prevent secondary SA infection in alveoli, thereby decreasing SA-induced lung injury and mortality. Future studies might investigate the extent to which ivacaftor is protective after SA infection has already initiated. Since ivacaftor is already in clinical use and has an excellent safety and tolerability profile (78), these findings may be translatable to patients.

In conclusion, our findings show that IAV lung infection induced reversal of normal alveolar liquid dynamics to cause airspace liquid absorption and inhibit AWL secretion. Thus, IAV infection abrogated a major mechanism of alveolar defense, leading to alveolar retention of inhaled SA and fatal SA-induced lung injury. Therapeutic rescue of AWL secretion was protective. These findings contribute new understanding of the role of alveolar liquid in health and lung injury. First, AWL secretion was critical to lung defense against inhaled SA, since loss of AWL secretion promoted alveolar SA infection. Second, although pathogen-induced lung injury is traditionally defined by gain of alveolar liquid (edema), our findings show that loss of alveolar liquid (AWL) was critical to IAV lung pathogenesis. Therapeutic approaches that restore AWL secretion in IAV-infected lungs may protect against fatal SA coinfection.

## Methods

### Experimental design.

Experiments were designed according to Animal Research: Reporting of *In Vivo* Experiments (ARRIVE) guidelines. Experimental units were single mice unless otherwise indicated. Mice used for in vivo studies were allocated to groups in a manner that ensured roughly equal mean mouse weight per group. Groups were mixed within cages, and assessments of breathing abnormalities and need for euthanasia were carried out by an investigator blinded to mouse groups. Outcome measures are indicated in figures and legends.

### Fluorophores.

We purchased calcein, acetoxymethyl ester (AM; 10 μM), calcein red-orange AM (10 μM), and tetramethylrhodamine (TRITC)-conjugated dextran from Thermo Fisher Scientific.

### Reagents.

Reagents were freshly constituted for experiments. Forskolin (20 μM) and amiloride (10 μM) were purchased from Selleckchem and CFTRinh-172 (20 μM) from MilliporeSigma. Ivacaftor was purchased from Selleckchem and reconstituted on delivery with DMSO (Thermo Fisher Scientific) before aliquoting and storage at –80°C. We prepared single doses of ivacaftor or vehicle within 1 hour of intraperitoneal (i.p.) administration. Doses were 40 mg/kg ivacaftor in 5% DMSO, 5% Tween-80, 40% PEG300 (all from Selleckchem), and 50% of 0.9% saline (Grifols) solution. Vehicle was the identical weight-based solution volume without ivacaftor.

### Solutions.

We purchased Ca^2+^- and Mg^2+^-containing Dulbecco’s PBS (DPBS) and Ca^2+^- and Mg^2+^-free PBS from Corning. Isolated lungs were perfused with HEPES-buffered vehicle of pH 7.4 and osmolarity 295 mOsm containing 150 mM Na^+^, 5 mM K^+^, 1 mM Ca^2+^, 1 mM Mg^2+^, and 10 mM glucose. Except where noted, fluorophores, reagents, and antibodies microinstilled in alveoli were dissolved or suspended in the same HEPES-buffered solution.

### Antibodies.

Antibodies were purchased from commercial vendors or academic institutions that provided antibody validation information. Allophycocyanin-conjugated monoclonal antibody (mAb) against CD11b (clone M1/70, catalog 14-0112-82, Thermo Fisher Scientific) was diluted 1:25 for alveolar microinstillation. Antibodies used for immunoblotting included mouse mAb against CFTR (clone A-3, catalog sc-376683, lot I2221, Santa Cruz Biotechnology) (43); mouse mAb against dephosphorylated CFTR (clone 570, catalog AB570, lot 570TJ20200526, University of North Carolina at Chapel Hill CFTR Antibody Distribution Program) (44, 45); and rabbit polyclonal antibody against actin (catalog A2066, lot 120878, MilliporeSigma). Secondary antibodies (LI-COR) included IRDye 800CW goat anti-mouse (catalog 925-32210, lot D01110-02) and IRDye 680LT goat anti-rabbit (catalog 925-68021, lot C90501-05). Antibodies were diluted in StartingBlock T20 Blocking Buffer (Thermo Fisher Scientific) and incubated with membranes as follows: CFTR mAb A-3 was diluted to 1:100 and incubated for 24–72 hours at 4°C; CFTR mAb 570 was diluted to 1:500 and incubated for 24 hours at 4°C; actin antibody was diluted to 1:2,000 and incubated for 1 hour at room temperature; and secondary antibodies were diluted to 1:10,000 and incubated for 40 minutes at room temperature.

### Viral preparation and inoculation.

Mouse-adapted IAV A/Puerto Rico/8/934 was propagated in 8-day-old embryonated chicken eggs (Charles River Laboratories), diluted in Ca^2+^- and Mg^2+^-containing DPBS, aliquoted, and stored at –80°C. IAV was intranasally instilled in anesthetized mice within 1 hour of thawing at a dose of 2,000 PFU in 20 μL or, for selected imaging experiments, 5,000 PFU in 50 μL.

### Bacterial strain, preparation, and inoculation.

SA was GFP-tagged strain USA300 LAC (SA^GFP^). Bacteria were stored at –80°C in 25% glycerol in autoclaved Luria-Bertani (LB) broth media (MP Biomedicals) and propagated on LB-agar plates containing chloramphenicol (10 μg/mL; MilliporeSigma). Plates were refreshed from frozen stock every 1–2 weeks. For experiments, single bacterial colonies were propagated in autoclaved LB media containing chloramphenicol (10 μg/mL) in a shaking incubator at 37°C and 200 rpm (New Brunswick Scientific) for 18 hours (stationary growth phase) or, for selected experiments, to OD_600nm_ = 1 (exponential growth phase). Bacteria were prepared for alveolar microinstillation or intranasal instillation, respectively, by dilution of 1 mL of culture in 500 μL or 1.3 mL in 300 μL of DPBS containing Ca^2+^ and Mg^2+^. Within 40 minutes of bacterial removal from the incubator, we instilled the bacteria-containing solution into lung alveolar airspaces by alveolar micropuncture or mice by intranasal instillation (30 μL to deliver 1 × 10^8^ CFU per mouse). For intranasal instillations, mice were rapidly anesthetized and instilled in pairs to ensure similarity of inocula across animals.

### Animals.

Mice were male Swiss Webster, purchased from Charles River Laboratories and Taconic Biosciences, 27–38 g, and 4–8 weeks old. We anesthetized mice with inhaled isoflurane (4%) for i.p. injections; or isoflurane and i.p. injections of ketamine (up to 100 mg/kg) and xylazine (up to 5 mg/kg) for intranasal instillations and surgical procedures. For surgeries, we injected the tail vein of anesthetized mice with heparin (50 U; Mylan), then exsanguinated the mice by cardiac puncture.

### Intranasal instillation and intraperitoneal injection.

Instillation and injection qualities were recorded on a 4-point scale at the time of instillation or injection by the performing investigator. In general, quality was considered acceptable if the instillation was recorded as 3 to 4 (i.e., little or no loss of instillate observed) or the injection was recorded as 4 (i.e., no injury or fluid leakage at the injection site).

### Isolated, blood-perfused lungs.

Using our reported methods (27), we cannulated the trachea, pulmonary artery, and left atrium of the heart of exsanguinated mice, then excised the heart, lungs, and cannulas en bloc. Lungs were inflated with room air through the tracheal cannula and perfused through the pulmonary arterial and left atrial cannulas at 0.4–0.6 mL/min with autologous blood in a solution of 4% dextran (70 kDa; Molecular Probes), 1% FBS (Gemini Bio-Products), and HEPES-buffered solution at pH 7.4, osmolality 320 mOsm/kg, and 37°C. We used in-line pressure transducers (ADInstruments) to maintain constant airway pressure 6 cm H_2_O via a continuous positive airway pressure machine (Philips Respironics) and pulmonary artery and left atrial pressures 10 and 3 cm H_2_O, respectively, via a roller pump (Ismatec). The lungs were positioned to enable micropuncture and imaging of the diaphragmatic surface of the right caudal lobe. Portions of the lung that were not used for micropuncture and imaging were covered with plastic wrap to prevent desiccation.

### Alveolar microinstillation.

We hand-beveled glass micropipettes (Sutter Instruments) to micropuncture single alveoli under bright-field microscopy, as we have done previously (27). Micropunctured alveoli were instilled with fluorophores, reagents, and antibodies in solution, resulting in their spread from the micropunctured alveolus to neighboring alveoli. For bacterial microinstillations, we prepared SA^GFP^-containing solutions as above, then microinstilled the solutions to deliver approximately 10^4^ CFU in 3 seconds of discontinuous microinstillation (79). Microinstillations were performed in 1–3 alveoli bordering each imaging field.

### Live lung imaging and analysis.

By our established methods (27), we viewed alveoli by confocal microscopy (LSM800, Zeiss) with a ×20 water immersion objective (NA 1.0; Zeiss) and coverslip. We used bright-field microscopy to randomly select regions of 30–50 alveoli for microinstillation and imaging. All images were acquired as single images using Zen (v2.6, Zeiss) and recorded as *Z*-sections. Analyzed images were 4–8 μm below the pleura. Optical thickness was 32–34 μm. Frame size was 512 × 512 pixels. We established laser, filter, pinhole, and detector settings at the beginning of each experiment to optimize alveolar fluorescence and avoid fluorescence saturation, then maintained the settings for the duration of the experiment. We confirmed absence of bleed-through between fluorescence emission channels. Images were analyzed using ImageJ (v2.0.0-rc-69/1.52n, NIH). Brightness and contrast adjustments were applied to individual color channels of entire images and equally to all experiment groups. We did not apply downstream processing.

### Airspace dextran fluorescence determination.

We used our established approach (27, 31) to microinstill airspaces with calcein-AM, then TRITC-conjugated dextran (70 kDa; 10 or 40 mg/mL in HEPES-buffered solution). Provided alveolar barrier function is intact, time-dependent loss of TRITC-dextran fluorescence indicates dilution by AWL secretion (31).

### Alveolar permeability determination.

To determine alveolar barrier properties, we added TRITC-conjugated dextran (20 kDa; 10 mg/mL) to the intact lung perfusate solution using our established methods (27).

### Immunoblot.

Using our reported methods (27), we cannulated the pulmonary artery of exsanguinated mice, then washed the lung vessels with ice-cold DPBS containing Ca^2+^ and Mg^2+^. The lungs were excised and snap-frozen in liquid nitrogen, then pulverized inside a specimen bag using a cold mortar and pestle. The pulverized lungs were mixed and incubated with RIPA buffer (Thermo Fisher Scientific) and Halt protease mix (Thermo Fisher Scientific) in a tissue grinder for 40 minutes on ice, then centrifuged for 20 minutes at 15,000 *g* and 4°C. We used the Pierce BCA Protein Assay Kit (Thermo Fisher Scientific), a plate reader (Molecular Devices) to standardize protein loading in Laemmli 2× Concentrate sample buffer (MilliporeSigma), and deionized water for gel electrophoresis (Invitrogen). Samples were heated to 37°C or 65°C for 5 or 10 minutes on a heating block before loading. Band densities were quantified using Image Studio (v5.2, LI-COR).

### Plasmid preparation, transfection, and instillation.

We transformed DH5α *E*. *coli* (New England Biolabs) with plasmid DNA via heat shock, then amplified and purified the plasmids using an EndoFree Plasmid Maxi Kit (Qiagen). By our established methods (27), we complexed plasmid DNA for A1440X mutant CFTR or non-mutant CFTR (gifts of Martina Gentzsch, University of North Carolina, Chapel Hill, North Carolina, USA) or vector (pcDNA 3.1, Invitrogen) with freshly extruded unilamellar liposomes (20 μg/μL; 100 nm pore size; DOTAP, Avanti Lipids) in sterile Opti-MEM (Gibco). For transfection, we administered 75 μg plasmid DNA per mouse by intranasal instillation.

### Survival assessment.

A blinded investigator assessed and recorded mouse weight, breathing score, and need for euthanasia in line with our IACUC-approved protocol. Euthanasia need was determined by a scoring system that included observations of mouse appearance, breathing, behavior, gait, and response to stimulation by cage top opening and placement on a narrow beam. Mice instilled with IAV alone were assessed at 24-hour intervals post-instillation for 4 days. Mice instilled with SA^GFP^ or PBS were assessed hourly for 6 hours after instillation, then at least every 12–24 hours for 3 days. Surviving mice were euthanized at the conclusion of experiments.

### Protein and leukocyte determinations in BAL fluid.

Using our reported methods (27), we cannulated the trachea of exsanguinated mice, then lavaged the lungs with 5 sequential instillations of 1 mL of ice-cold, Ca^2+^-free PBS. For total protein determinations, we centrifuged the first aliquot of BAL fluid return (minimum volume 0.78 mL) for 10 minutes at 400 *g* and 4°C, then centrifuged the supernatant again for 20 minutes at 15,000 *g* and 4°C. Total protein was quantified using the Pierce BCA Protein Assay Kit (Thermo Fisher Scientific). For leukocyte determinations, BAL samples were pooled on a per-mouse basis and centrifuged for 10 minutes at 500 *g*. The resuspended cells were incubated for 10 minutes in Türk’s solution (MilliporeSigma), then counted using a hemacytometer.

### Lung wet weight to body weight ratio and extravascular lung water quantifications.

Lung wet weight and extravascular lung water (EVLW) were quantified in the same experiment. Body weight was recorded in anesthetized mice at the time of lung excision in untreated mice or at the time of IAV instillation in coinfected mice. We used our established methods (27) to exsanguinate anesthetized mice by cardiac puncture, then excised and weighed the lungs. Blood-free EVLW content was quantified by the method of Selinger and colleagues (80), which we have used previously (27). Lungs were cut with scissors, then processed using a handheld tissue homogenizer. Hemoglobin content was determined by spectrophotometry (Molecular Devices) using hemoglobin standards and a solution of Drabkin’s reagent and Brij L23 (MilliporeSigma). Homogenate, supernatant, and blood samples were dried for 24 hours in a vacuum oven at 57°C and –5 mmHg. Total EVLW content was normalized to body weight to account for increases of lung dry weight due to extravasated protein (81).

### Viral and bacterial counts.

For bacterial quantifications, we collected blood by cardiac puncture, obtained BAL fluid by the methods above, and excised the spleen, liver, and lungs from exsanguinated mice using our reported methods (27). Organs were mechanically homogenized by crushing in a specimen bag and diluted in 1 mL of DPBS containing Ca^2+^ and Mg^2+^. SA^GFP^ CFU was quantified by serial dilutions on chloramphenicol-containing LB agar plates. For viral quantifications, lungs were homogenized in homogenizer tubes (Benchmark Scientific) in 500 μL of Ca^2+^-free PBS. IAV PFU was determined by plaque assay (82). Briefly, homogenized lungs were 10-fold serially diluted starting from 1:10 dilution and added to a confluent monolayer of Madin-Darby canine kidney cells (line CCL-34, ATCC) for 1 hour at 37°C and 5% CO_2_ with gentle rocking. The inoculum was removed, and the cells were overlaid with a solution composed of 1% agar (Oxoid) and 2× minimal essential medium supplemented with 1% diethyl-aminoethyl-dextran, 5% NaHCO_3_, and 1 μg/mL tosylamide-2-phenylethyl chloromethyl ketone–treated trypsin. Cell-containing plates were incubated for 48 hours at 37°C and 5% CO_2_, then fixed in 10% formaldehyde overnight. Plaques were visualized by immune labeling with mAb against HT-103 (gift of Thomas Moran, Icahn School of Medicine at Mount Sinai, New York, New York, USA), HRP-conjugated anti-mouse secondary detection antibody, and TrueBlue substrate (KPL-Seracare).

### Statistics.

Statistics are indicated in figures and legends. In general, paired comparisons were analyzed by *t* tests, multiple comparisons by ANOVA with posthoc testing, and survival comparisons by log rank testing. We considered *P* values less than 0.05 to be statistically significant. Data were analyzed and figures were prepared using Microsoft Excel, StatPlus:mac Pro (version v7, AnalystSoft Inc.), and SigmaPlot (version 14.5, Systat). In rare instances, mice were excluded from analyses if instillations or tissue collections failed to meet our laboratory’s standards for quality. Reasons for exclusion included death within 5 minutes of SA^GFP^ instillation (1 mouse), persistent gait abnormalities or bleeding at i.p. injection sites (3 mice), and trachea rupture during BAL fluid collection (1 mouse). Exclusion decisions were made in an identical manner for all groups and based on quality assessments performed while experiments were ongoing, thus prior to data analysis.

### Study approval.

The Institutional Animal Care and Use Committees of the Icahn School of Medicine at Mount Sinai and Columbia University Medical Center approved the animal procedures.

### Data availability.

Data are available in the Supporting Data Values file.

## Author contributions

JLH designed the study, wrote the manuscript, and was responsible for the overall project. JLH, ST, ACD, DC, SS, SKLM, KS, SH, and RR contributed to data collection and data analysis. JLH, ST, ACD, DC, SS, SKLM, AJM, CJB, MS, and JB contributed to the experimental design and interpretation of results. All authors edited the manuscript. The order of the co–first authors was determined based on ST’s greater contribution to writing and figure composition.

## Supplementary Material

Supplemental data

Supporting data values

## Version 1 08/15/2023

In-Press Preview

## Version 2 10/02/2023

Electronic publication
