# Supplementary material for: Rescue of alveolar wall liquid secretion blocks fatal lung injury due to influenza-staphylococcal coinfection
Source: J Clin Invest. 2023 Oct 2;133(19):e163402. doi: 10.1172/JCI163402 (PMC10541650; doi:10.1172/JCI163402)
Supplement: Supplemental data [file jci-133-163402-s155.pdf]

Supplemental Figure 1

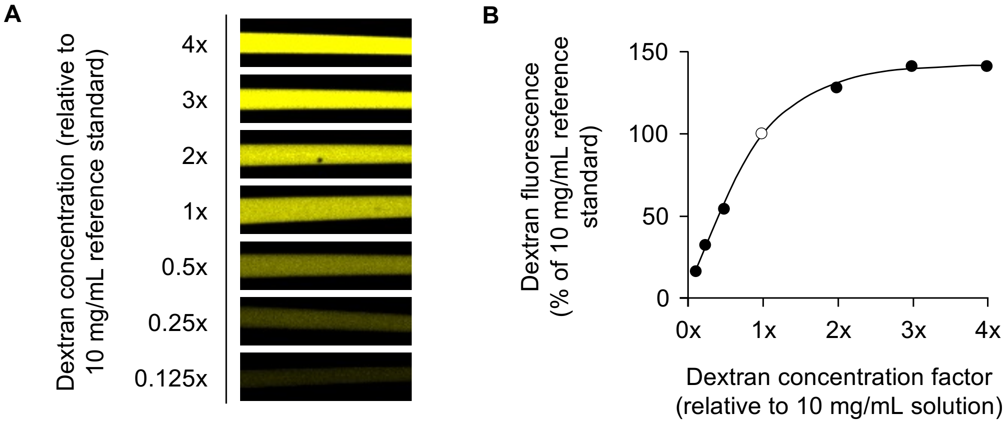

Supplemental Figure 1. Dextran fluorescence calibration in glass micropipettes. Confocal images (**A**) and plot (**B**) show the relationship between concentration and fluorescence intensity of tetramethylrhodamine (TRITC)-conjugated dextran (70 kD) in aqueous solution in glass micropipettes. *Open circle* (B) indicates the 10 mg/mL reference standard. *Line* calculated by polynomial regression ($P < 0.05$).

Supplemental Figure 2

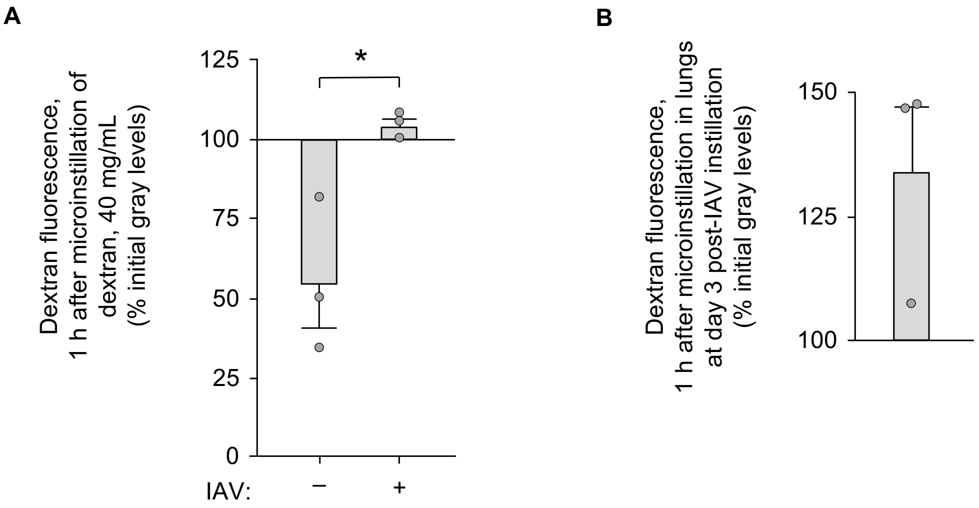

Supplemental Figure 2. Experiments related to AWL secretion. (**A-B**) Group data show results of imaging of live, intact, perfused mouse lungs and quantify change of TRITC-labeled dextran fluorescence after dextran microinstillation into alveolar airspaces. In A, mice were untreated (–) or intranasally-instilled with IAV for 24 h (+) as indicated. In B, lungs were excised for imaging on day 3 after IAV instillation. Circles indicate *n* and each represent one mouse in which change of dextran fluorescence was quantified in imaging fields of at least 30 alveoli. Bars: mean ± SEM. In A, *$P < 0.05$ by two-tailed *t* test.

Supplemental Figure 3

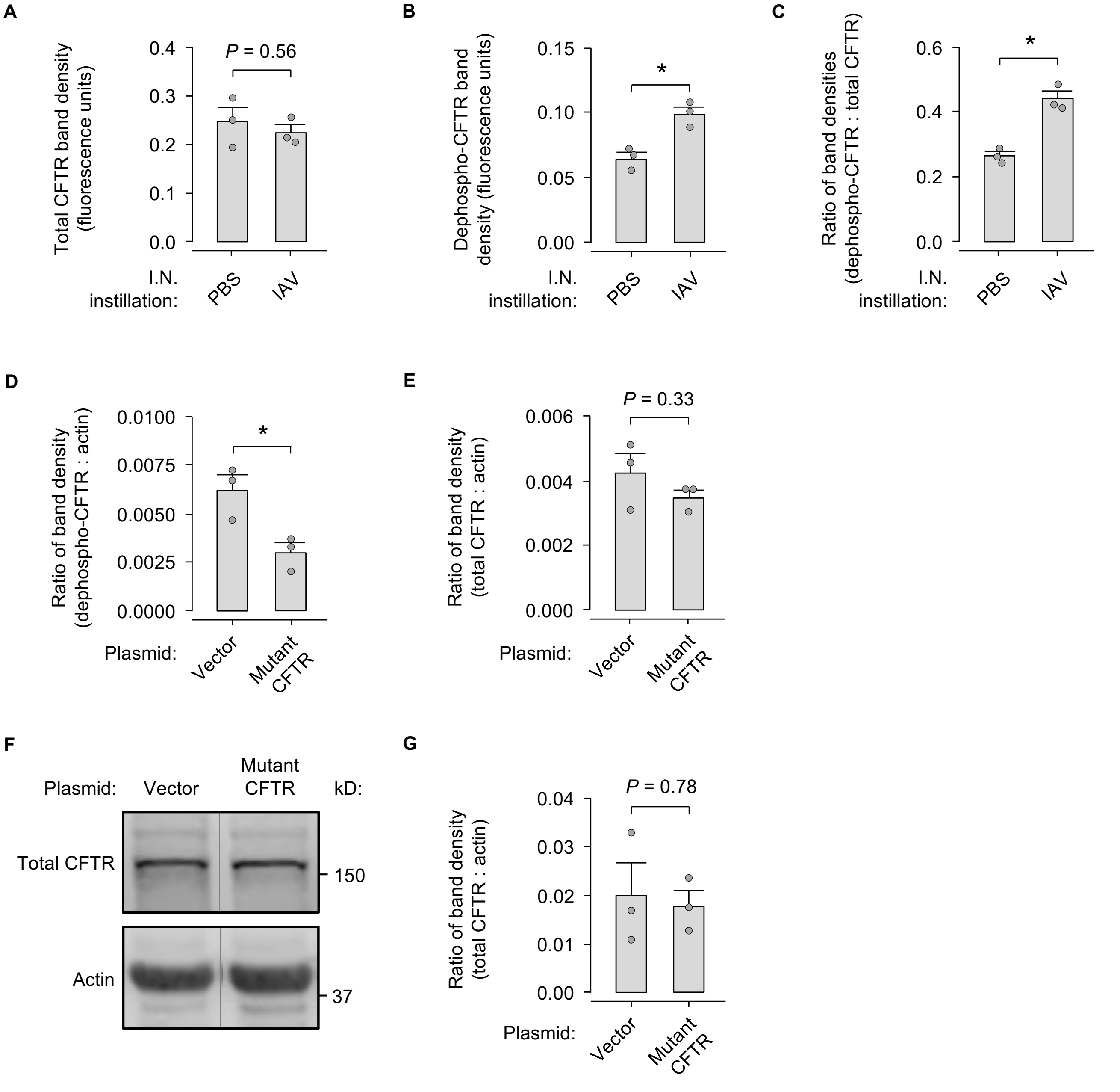

Supplemental Figure 3. Immunoblot and band densitometry of total and dephosphorylated CFTR protein bands. (**A-E**) Group data in A-C and D-E show band densities quantified from immunoblot data shown in representative images in Figures 4A and 4E, respectively. Lungs from mice intranasally-instilled with PBS (A-C) or IAV (A-E) were excised at 24 h post-instillation and homogenized. For D-E, mice were pretreated with intranasal instillation of liposome-complexed plasmid DNA encoding plasmid vector or A1440X mutant CFTR protein, as indicated, at 24 h prior to IAV instillation. Band densities were quantified without (A-C) or with (D-E) normalization to actin band density. Circles indicate *n* and each represent lungs of one mouse. Bars: mean ± SEM; **P* < 0.05 by two-tailed *t* test. (**F-G**) Representative images (F) and group data of band densitometry (G) show immunoblot results of whole lung lysate. Mice were intranasally instilled with vector or A1440X mutant CFTR plasmid at 48 h prior to lung excision for immunoblot. Lanes were run on the same gel but were noncontiguous. Actin-probed membranes are not shown. Circles indicate *n* and each represent lungs of one mouse. Bars: mean ± SEM; **P* < 0.05 by two-tailed *t* test.

Supplemental Figure 4

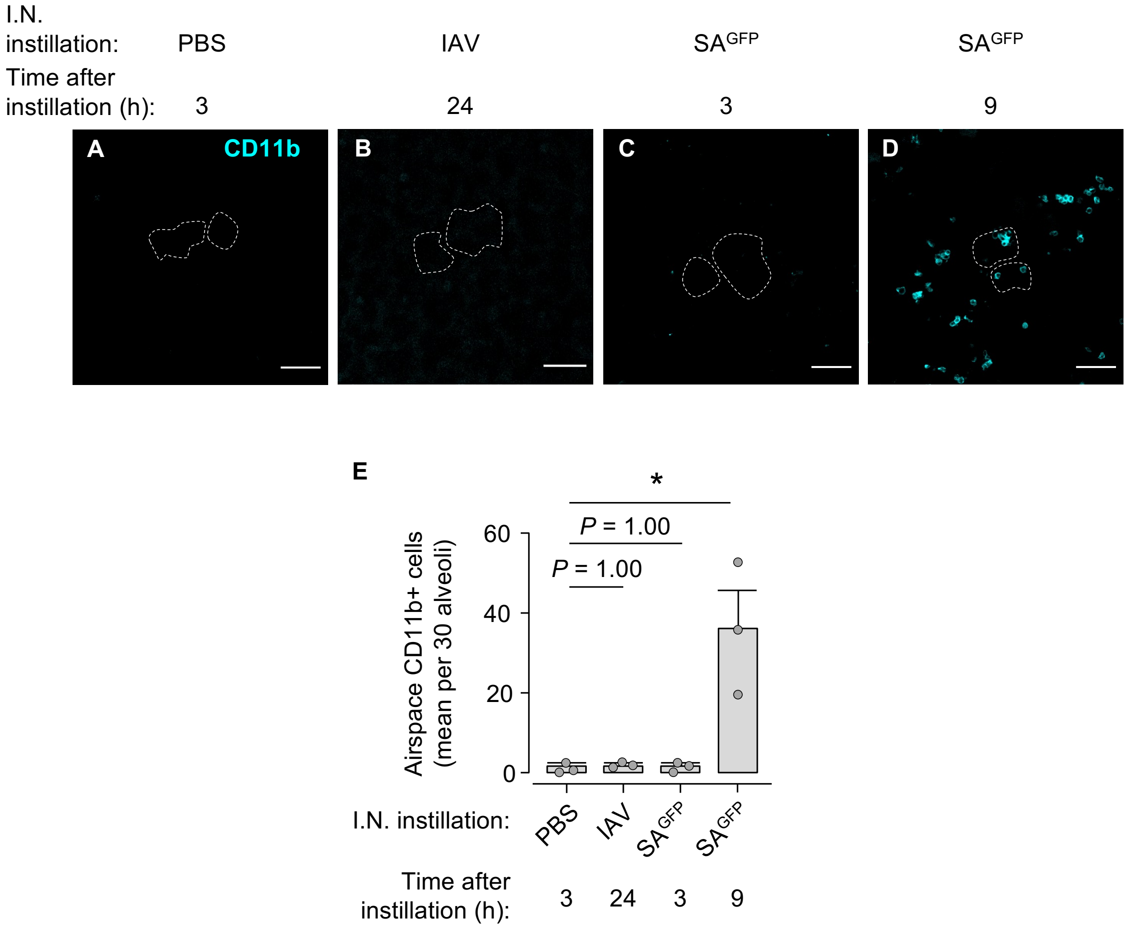

Supplemental Figure 4. Leukocytes in airspaces of live alveoli after intranasal pathogen instillation. Confocal images (**A-D**) and group data (**E**) show airspace fluorescence of allophycocyanin-conjugated CD11b antibody after alveolar antibody microinstillation in live, intact, blood-perfused mouse lungs. *Dotted lines* delineate example alveolar walls (fluorescence not shown). Imaging fields contain at least 30 alveoli each. Prior to imaging, mice were pretreated with intranasal instillation of PBS, IAV, or SA^{GFP} as indicated, then the lungs were excised for imaging at the indicated time post-instillation. After antibody microinstillation, alveoli were microinstilled with HEPES-based buffer to remove non-specific antibody fluorescence. Note, CD11b fluorescence is apparent in alveolar airspaces only in lungs excised at 9 h after intranasal SA^{GFP} instillation. For group data in E, circles indicate *n*, each represent one mouse, and were generated by quantifying the mean number of CD11b+ cells per imaging field of at least 30 alveoli. Bars: mean ± SEM; **P* < 0.05 versus left bar by ANOVA with post hoc Tukey testing. Scale bars: 50 μm.

Supplemental Figure 5

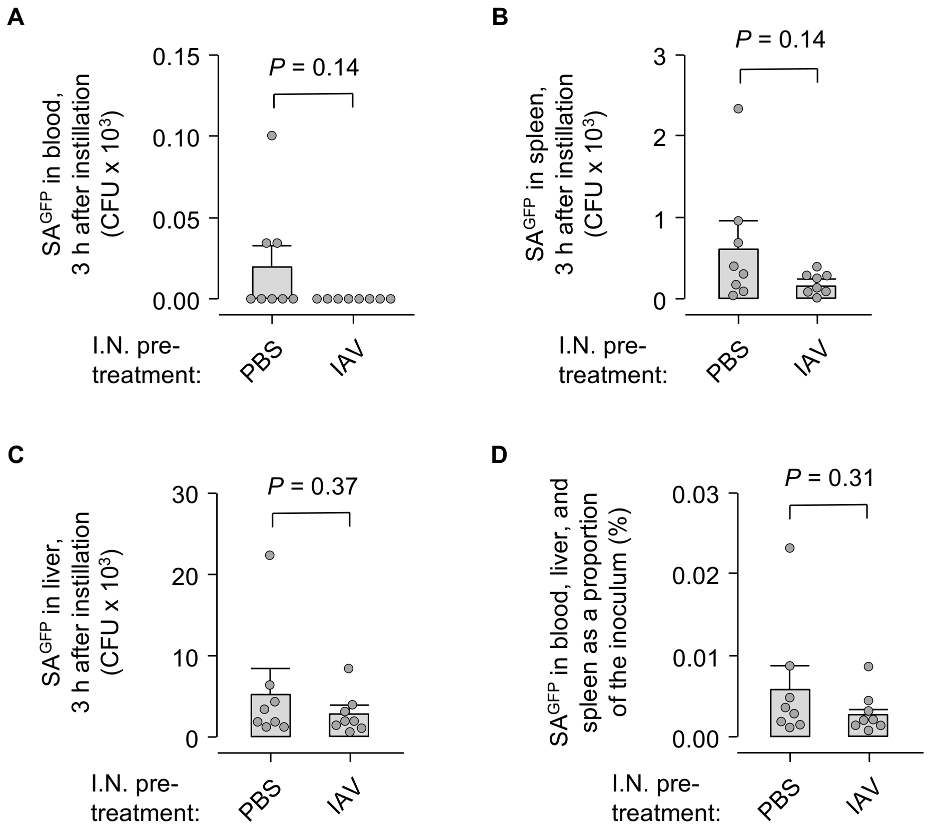

Supplemental Figure 5. SA^{GFP} quantifications in PBS- and IAV-infected mice. (A-D) Mice were pretreated with intranasal instillation of IAV or PBS as indicated, then intranasally instilled with SA^{GFP} 24 h later. Group data show SA^{GFP} quantifications in the indicated fluids and organs. Circles indicate *n* and each represent one mouse. Bars: mean ± SEM; **P* < 0.05 by two-tailed *t* test.

Supplemental Figure 6

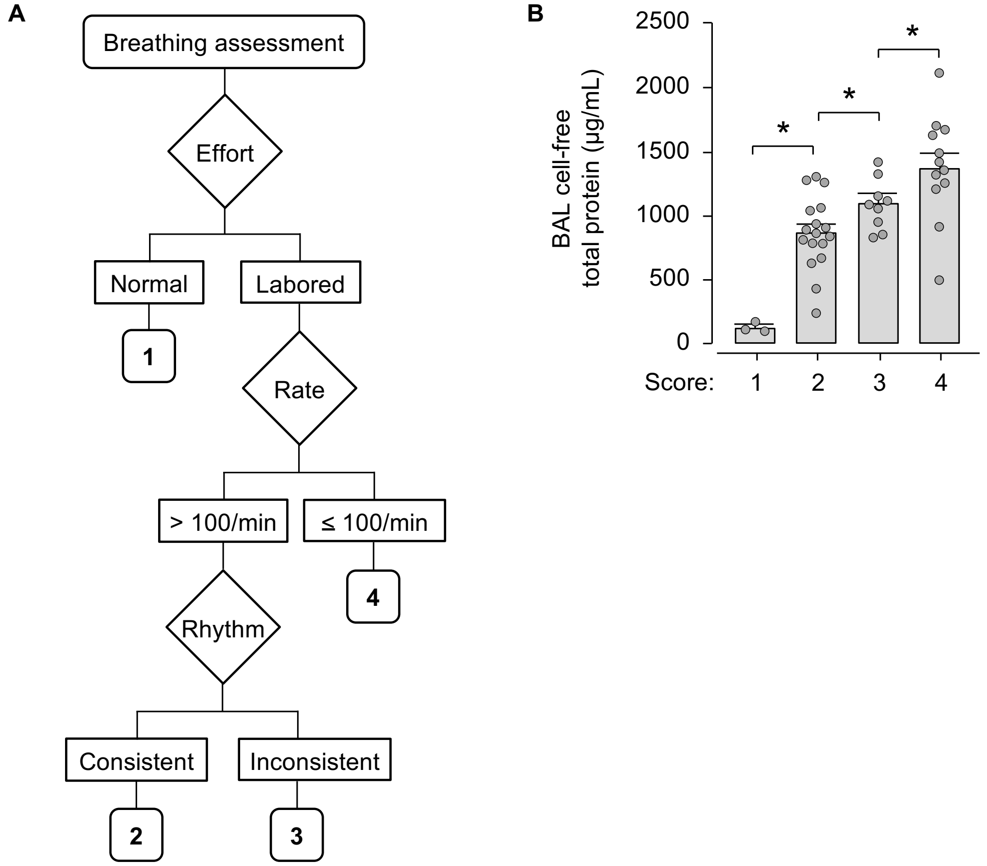

Supplemental Figure 6. Breathing score assessment. For breathing score assessments, investigators blinded to mouse group assigned scores to mice on a 1-4 scale using the flow diagram indicated in **A**. Group data in **B** show the correlation between breathing scores and total protein content in BAL fluid of pathogen-instilled mice. Circles (B) indicate *n* and each represent BAL protein content in one mouse. To generate the group data, we assigned breathing scores to mice infected with IAV, SA^{GFP}, or both, then plotted the relationship between score and protein content. Bars: mean ± SEM; *$P < 0.05$ by two-tailed *t* test as indicated.

Supplemental Figure 7

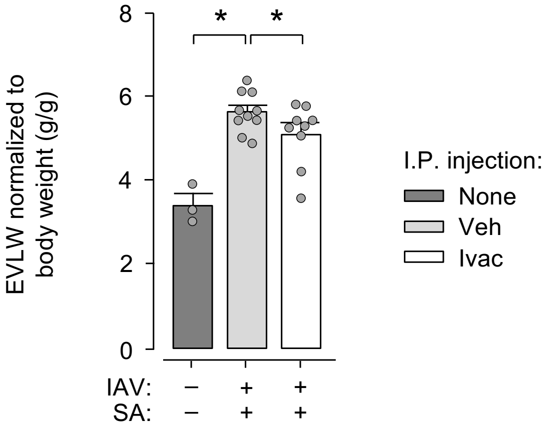

Supplemental Figure 7. AWL rescue therapy protects against acute lung injury in coinfected mice. As indicated in the figure, mice were untreated or treated sequentially with: (1) intranasal instillation of IAV; (2) intraperitoneal (*I.P.*) injection of vehicle (*veh*) or ivacaftor (*ivac*) at 6 h; and (3) intranasal instillation of SA^{GFP} at 24 h. In treated mice, lungs were excised for quantification of blood-free extravascular lung water (*EVLW*) 24 h after SA^{GFP} instillation. Bars indicate mean ± SEM; circles indicate *n* and each represent data from one mouse; *$P < 0.05$ as indicated by *t* test.

Supplemental Figure 8

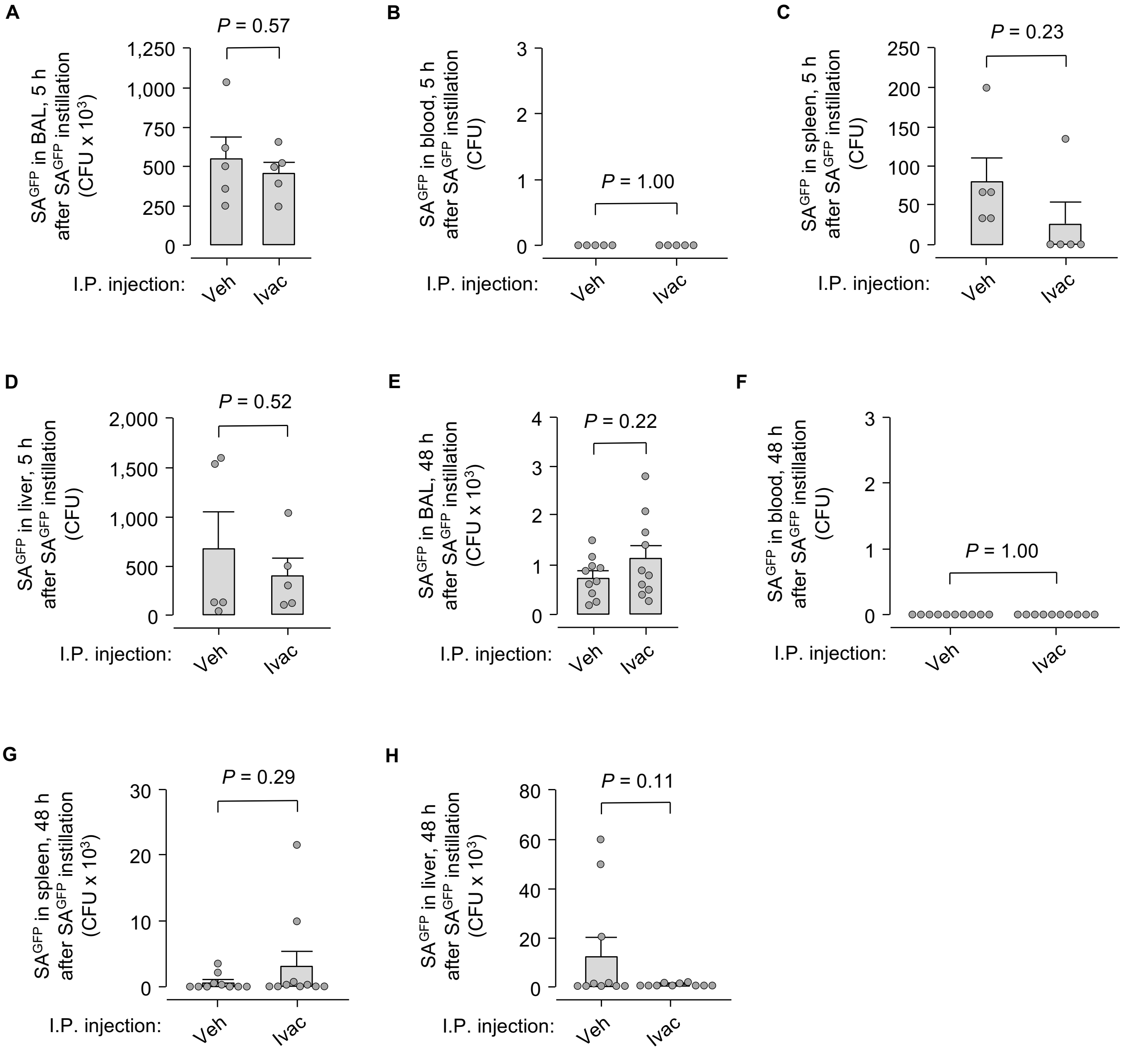

Supplemental Figure 8. SA^{GFP} quantifications in coinfected mice. (A-H) Group data show SA^{GFP} quantifications in the indicated fluids and organs. Mice were treated sequentially with: (1) intranasal instillation of IAV; (2) intraperitoneal (*I.P.*) injection of vehicle (*veh*) or ivacaftor (*ivac*) at 6 h; and (3) intranasal instillation of SA^{GFP} at 24 h. For E-H, mice were additionally treated with: (4) I.P. injection of vehicle or ivacaftor at 30 h; and (5) I.P. injection of vehicle or ivacaftor at 54 h. Fluids and organs were collected at 5 h (A-D) or 48 h (E-H) after SA^{GFP} instillation. Circles indicate *n* and each represent one mouse. Bars: mean ± SEM; $^*P < 0.05$ by two-tailed *t* test.

Supplemental Figure 9

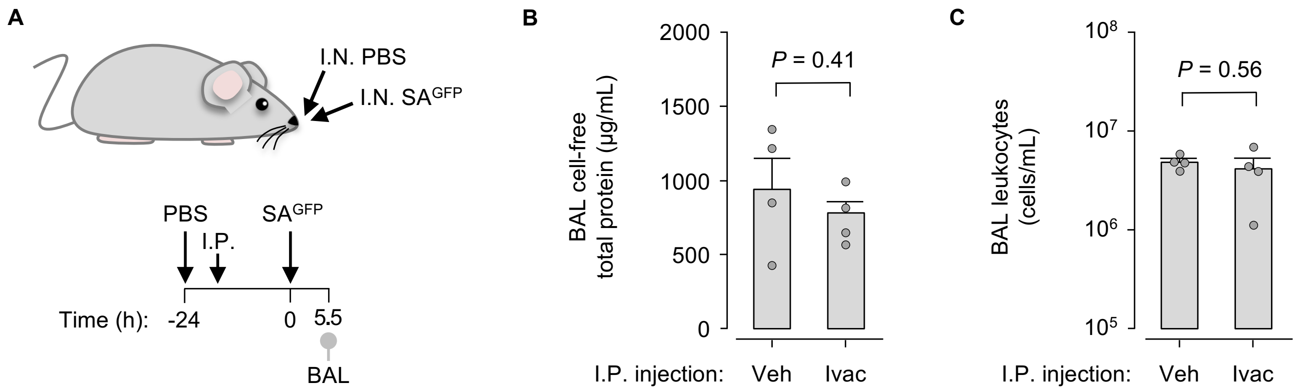

Supplemental Figure 9. AWL rescue therapy does not affect early outcomes of SA lung infection in mice without IAV infection. (**A-C**) Experimental design (A) for group data shown in B-C shows timing of intranasal (*I.N.*) instillations, intraperitoneal (*I.P.*) injections, and BAL fluid collection for quantification of total protein (B) and leukocyte (C) content. In B-C, bars indicate mean ± SEM; circles indicate *n* and each represent data from one mouse; *P* value as indicated by two-tailed *t* test. BAL content of protein and leukocytes were quantified using the same BAL fluid specimen.
